# The optimisation of deep neural networks for segmenting multiple knee joint tissues from MRIs

**DOI:** 10.1016/j.compmedimag.2020.101793

**Published:** 2020-12

**Authors:** Dimitri A. Kessler, James W. MacKay, Victoria A. Crowe, Frances M.D. Henson, Martin J. Graves, Fiona J. Gilbert, Joshua D. Kaggie

**Affiliations:** a Department of Radiology, University of Cambridge, Cambridge, United Kingdom; b Norwich Medical School, University of East Anglia, Norwich, United Kingdom; c Cambridge University Hospitals NHS Foundation Trust, Addenbrooke’s Hospital, Cambridge, United Kingdom; d Department of Veterinary Medicine, University of Cambridge, United Kingdom

**Keywords:** Magnetic resonance imaging (MRI), Musculoskeletal, Image segmentation, Convolutional neural network (CNN), Generative adversarial network (GAN)

## Abstract

Automated semantic segmentation of multiple knee joint tissues is desirable to allow faster and more reliable analysis of large datasets and to enable further downstream processing e.g. automated diagnosis.

In this work, we evaluate the use of conditional Generative Adversarial Networks (cGANs) as a robust and potentially improved method for semantic segmentation compared to other extensively used convolutional neural network, such as the U-Net. As cGANs have not yet been widely explored for semantic medical image segmentation, we analysed the effect of training with different objective functions and discriminator receptive field sizes on the segmentation performance of the cGAN. Additionally, we evaluated the possibility of using transfer learning to improve the segmentation accuracy. The networks were trained on i) the SKI10 dataset which comes from the MICCAI grand challenge “Segmentation of Knee Images 2010″, ii) the OAI ZIB dataset containing femoral and tibial bone and cartilage segmentations of the Osteoarthritis Initiative cohort and iii) a small locally acquired dataset (Advanced MRI of Osteoarthritis (AMROA) study) consisting of 3D fat-saturated spoiled gradient recalled-echo knee MRIs with manual segmentations of the femoral, tibial and patellar bone and cartilage, as well as the cruciate ligaments and selected peri-articular muscles. The Sørensen–Dice Similarity Coefficient (DSC), volumetric overlap error (VOE) and average surface distance (ASD) were calculated for segmentation performance evaluation.

DSC ≥ 0.95 were achieved for all segmented bone structures, DSC ≥ 0.83 for cartilage and muscle tissues and DSC of ≈0.66 were achieved for cruciate ligament segmentations with both cGAN and U-Net on the in-house AMROA dataset. Reducing the receptive field size of the cGAN discriminator network improved the networks segmentation performance and resulted in segmentation accuracies equivalent to those of the U-Net. Pretraining not only increased segmentation accuracy of a few knee joint tissues of the fine-tuned dataset, but also increased the network’s capacity to preserve segmentation capabilities for the pretrained dataset.

cGAN machine learning can generate automated semantic maps of multiple tissues within the knee joint which could increase the accuracy and efficiency for evaluating joint health.

## Highlights

• A conditional Generative Adversarial Network (cGAN) is optimised for automated segmentation of multiple knee joint tissues.
• This work quantitatively compares the cGAN with the widely used U-Net approach for semantic image segmentation.
• Transfer learning for improved segmentation performance of an in-house dataset is explored.
• Pretraining on the SKI10 / OAI ZIB datasets increased segmentation accuracy and preserved segmentation capabilities of the previous training.

## 1 Introduction

Osteoarthritis (OA) is a degenerative disease involving the entire synovial joint (Goldring et al., 2017; Hunter and Eckstein, 2009; Martel-Pelletier et al., 2016). Important risk factors for the development of OA include age, muscle weakness, abnormal joint loading due to joint malalignment or overloading (obesity, high impact sport), and injury to the menisci and ligaments (Ismail and Vincent, 2017; Lohmander et al., 2007; Martel-Pelletier et al., 2016). Distinctive hallmarks of OA include the progressive destruction of articular cartilage structure and alterations in the surrounding joint tissues, including bone, meniscus, ligament and peri-articular muscle. Magnetic resonance imaging (MRI) is a commonly used tool to evaluate clinical abnormalities of the knee (Blumenkrantz and Majumdar, 2016). Morphological changes due to OA are well demonstrated with MRI (Benhamou et al., 2001; Hunter et al., 2015; MacKay et al., 2018; Neogi et al., 2013; Wise et al., 2018). Tissue specific masks of the knee joint can be useful for the analysis of OA, especially as automated tools continue to be developed and validated (Bindernagel et al., 2011; Deniz et al., 2018; Lee et al., 2014; Liu et al., 2017; Ng et al., 2006; Patel and Singh, 2018; Seim et al., 2010; Shan et al., 2014; Shrivastava et al., 2014; Swanson et al., 2010; Xia et al., 2013; Zhou et al., 2016).

For both clinical and research usage, a significant amount of time is spent manually segmenting images to designate tissue-specific regional masks, also known as regions-of-interest (ROIs). Image masking remains a very significant challenge within medical imaging due to heterogeneity in organ appearance and disease progression and presentation. The segmentation of neighbouring soft tissues such as the cruciate ligaments, cartilages and muscles in the knee joint which have similar image intensities (and therefore poor contrast resolution) is an especially demanding task. ROIs can be generated through manual or semi-manual delineation by a trained reader, or they may be generated automatically using signal thresholding (Swanson et al., 2010), shape (Bindernagel et al., 2011; Seim et al., 2010), atlas (Lee et al., 2014; Shan et al., 2014), or derive from region based (Ng et al., 2006; Patel and Singh, 2018; Shrivastava et al., 2014) approaches, as well as with machine learning approaches (Deniz et al., 2018; Liu et al., 2017; Xia et al., 2013; Zhou et al., 2016). Machine learning methods include unsupervised learning, such as k-means clustering, which segments based on spatial clusters of similar signal intensities in an image (Ng et al., 2006; Patel and Singh, 2018; Shrivastava et al., 2014), or supervised learning by training the algorithm on image masks that have been obtained from any previous masking technique (Deniz et al., 2018; Liu et al., 2017; Xia et al., 2013; Zhou et al., 2016). The number of high-quality label maps for supervised learning is typically very small, and the performance of a machine learning network trained on a low number of data is limited due to the lack of heterogeneity of images presented during training. Transfer learning may be used to mitigate this by pretraining a network on a large dataset with different but related similarities to the actual task, followed by network refinement on the small dataset (Shie et al., 2015).

Convolutional neural networks (CNNs), in particular U-Nets (Ronneberger et al., 2015), have demonstrated their capability to automate the segmentation of musculoskeletal MRIs (Liu et al., 2017; Norman et al., 2018). Nevertheless, a drawback of this approach with CNNs is that they usually use pixel-wise measures such as the absolute (L1) or square (L2) error loss which can be non-optimal for image data, and, in the case of L2, result in blurry boundaries (Pathak et al., 2016). In contrast, generative adversarial networks (GANs) (Goodfellow et al., 2014) learn a similarity measure (feature-wise metric) that adapts to the training task by implementing two competing, or adversarial, neural networks. During adversarial training, one network focusses on image discrimination and guides a second network which focusses on image generation to create “real” images that have a data distribution indistinguishable from the training data distribution. The generator and discriminator are trained simultaneously and competitively in a mini-max game while convergence is achieved when the Nash equilibrium is reached, i.e. no network can improve through further training if one remains unchanged (Zhao et al., 2017).

Conditional GANs (cGANs) modify the GAN approach to learn image-to-image mappings (Goodfellow et al., 2014; Isola et al., 2017). In comparison to traditional GANs that learn a mapping from random noise to a generated output, cGANs learn a mapping from an observed variable, for example an image to generate an output, such as a label map (Goodfellow et al., 2014; Isola et al., 2017). cGANs have been used to produce image labels for neurological (Rezaei et al., 2017), cardiac (Dou et al., 2018), abdominal (Huo et al., 2018), respiratory (Chen et al., 2018) and musculoskeletal imaging (Liu, 2018, Gaj et al., 2019). (Liu, 2019) used unpaired image-to-image translation with a method called cycle-consistent generative adversarial network (CycleGAN) to perform semantic image segmentation of femorotibial cartilage and bone of the knee joint of unlabelled MRI datasets. The “pix2pix” framework is one cGAN approach that has demonstrated segmentation capability (Isola et al., 2017). Semantic segmentation with cGANs, particularly those combining U-Net generators and Markov Random Field discriminators (patch-based discriminators), is relatively unexplored. The method has previously been performed for semantic segmentation of the brain (Rezaei et al., 2017). In (Gaj et al., 2019), a cGAN was used for semantic segmentation of knee cartilage and meniscus but with an image-wise discriminator rather than a patch-wise discriminator.

The aim of this study was to implement and evaluate a cGAN for automated semantic segmentation of multiple joint tissues from MR images: the femoral, tibial and patellar bones and cartilage surfaces; the cruciate ligaments; and two selective muscles, the medial vastus and gastrocnemius. Our essential contributions are summarised as followed:

1 Implementation of a cGAN based on the “pix2pix” framework introduced by (Isola et al., 2017) using a U-Net generator and a patch-based discriminator for automatic segmentation of multiple knee joint tissues. As far as we know, cGANs have not previously been used for semantic segmentation of the patellar bone and cruciate ligaments, as well as muscles of the knee joint.
2 Evaluating the segmentation performance of the cGAN with different objective functions by combining the cGAN loss with different pixel-wise error losses and modifying the weighting hyperparameter between the cGAN loss and pixel-wise error loss.
3 Assessing the choice of the generator depth and discriminator receptive field size on the performance of the cGAN for multi-tissue segmentation.
4 Quantitative comparison of the cGAN approach with the well-known U-Net approach.
5 Exploring the use of transfer learning for improved segmentation performance of both cGAN and U-Net.

## 2 Material and methods

### 2.1 Image datasets

Three image datasets were used for network training and testing; the publicly available SKI10 and OAI ZIB datasets, consisting of 100 and 507 labelled knee MRs, respectively, and a locally acquired dataset of ten segmented knee MRs (Advanced MRI of Osteoarthritis (AMROA) study).

#### 2.1.1 SKI10

The “Segmentation of Knee Images 2010″ (SKI10) dataset (Heimann et al., 2010), consists of approximately 90 % 1.5 T and 10 % 3.0 T sagittal MR images using multiple system vendors – GE, Siemens, Philips, Toshiba, and Hitachi. The sequences were varied and included both gradient echo and spoiled gradient echo sequences, commonly with fat suppression. The images were segmented on a slice-by-slice basis by experts from Biomet, Inc., initially through intensity thresholds and thereafter with manual editing. One hundred 3D image datasets of the SKI10 challenge were provided with semi-manual masks of femoral and tibial cartilage and bone. In our study, 70 datasets were used for network training and 30 for network testing.

#### 2.1.2 OAI ZIB

The OAI ZIB dataset (Ambellan et al., 2019) is comprised of segmentations of femoral and tibial cartilage and bone of 507 MR imaging volumes from the publicly available Osteoarthritis Initiative dataset (The Osteoarthritis Initiative, 2020). The MR images were acquired on Siemens 3 T Trio systems using a 3D double echo steady state (DESS) sequence with water excitation. Outlines of femoral and tibial bone and cartilage were generated using a statistical shape model (Seim et al., 2010) with manual adjustments performed by experts at Zuse Institute Berlin. The OAI ZIB data covers all degrees of OA (KL 0–4), with more cases having severe OA (KL ≥ 3) (Ambellan et al., 2019). As with the SKI10 dataset, we split the dataset in 70 % (355) for network training and 30 % (152) for testing.

#### 2.1.3 AMROA

The locally acquired participant cohort consisted of ten subjects: five healthy volunteers and five patients with mild-to-moderate OA. The patients followed at least one subset of American College of Rheumatology criteria for OA and were recruited between April 2017 to April 2018 (Table 1). The healthy volunteers were approximately matched to OA patients for age, sex, and body mass. Network training was performed on data from four subjects with OA and four healthy subjects. Two individuals (one with OA and one healthy) were used as a unique set for test measurements. The number of test individuals was chosen such that roughly 80 % of the data could be used for training. Ethical approval was obtained from the National Research Ethics Service, and all subjects provided written informed consent before participation.

**Table 1:** Participant characteristics showing the mean age, number of males/females (M/F), average body-mass-index (BMI), Kellgren-Lawrence (KL) osteoarthritis score and the number of training/testing set images of the locally acquired dataset. Additionally, the number of participants (N) and training/testing set images of the SKI10 and OAI ZIB datasets are given.

Dataset	Variable	Training Set	Testing Set
Local	N	8	2
	Images	806	171
	Mean Age (years)	53	52
	Sex (M/F)	5/3	0/2
	Mean BMI (kg/m^2^)	27.8	27.7
	KL (0/2/3)	4/1/3	1/1/0
SKI10	N	70	30
	Images	6,133	2,626
OAI ZIB	N	355	152
	Images	43,814	18,517

The source images (Fig. 2A) for each subject were 3D fat-saturated spoiled gradient recalled-echo (3D-FS SPGR) images and were acquired on a 3.0 T MRI system (MR750, GE Healthcare, Waukesha, WI, USA) using an 8-channel transmit/receive knee coil (InVivo, Gainesville, FL, USA). The 3D-FS SPGR sequence parameters were: field-of-view = 150 × 128 × 136 mm^3^, matrix size = 512 × 380 × 136 zero-fill interpolated to 512 × 512 × 136, voxel size = 0.29 × 0.29 × 1.0 mm^3^, TR = 12.5 ms, TE = 2.4 ms, flip angle = 25°, coil acceleration factor (ASSET) = 2, partial Fourier phase encoding = 0.5 (half-NEX), bandwidth = ±11.9 kHz, with fat-suppression.

Semi-manual segmented masks (Fig. 2A) of the patella, tibia, and femur bones as well as of their respective surrounding patellar, tibial and femoral cartilages (Fig. 2b) were created from the 3D-FS SPGR images by a musculoskeletal radiologist with 8 years’ experience, using the Stradwin software v5.4a (University of Cambridge Department of Engineering, Cambridge, UK, now freely available as ‘StradView’ at http://mi.eng.cam.ac.uk/Main/StradView/) (MacKay et al., 2020). Additionally, masks of the vastus medialis and medial head of gastrocnemius muscles were created. This semi-manual segmentation pipeline consists of sparse manual contour generation (every 2nd-5th sagittal image/2−5 mm) followed by automatic surface triangulation using the regularised marching tetrahedra method. Volume preserving surface smoothing allows creation of an accurate segmentation from relatively sparse manual contours (Treece et al., 1999). Manual segmentations of the anterior cruciate ligament (ACL) and posterior cruciate ligament (PCL) were created on the 3D-FS SPGR images using ITK SNAP (Yushkevich et al., 2006) by a radiologist with 3 years’ experience.

### 2.2 Training data and masking

Each of the major structures were given a separate image value, i.e., colour, in the segmentation mask, such that the network determined the unique weights to generate a similar regional colour-value from an MR image. On a 256-bit colour-scale, the three bones were stored in the blue colour channel where the femur colour code was 50, tibia was 100, and patella was 150. The cartilages were stored in the green colour channel where the femoral cartilage colour code was 50, the tibial was 100 and the patellar was 150. Additionally, for the AMROA dataset, the muscles were stored in the red colour channel with the medial vastus muscle code set to 100 and the medial gastrocnemius muscle colour code set to 200. The ACL mask was stored in the blue colour channel and the PCL in the green colour channel with both colour codes set to 200.

The MRIs and image masks were converted from the DICOM and NIFTI formats (Larobina and Murino, 2014), respectively, to a common image format (Portable Network Graphics, PNG) before training. Noise-only images were not used for training or testing, as training a network to fit against zero-valued masks results in a poor constraint. After network training, a tissue- / region-specific Boolean mask was created on the predicted test images by removing prediction values outside of ±20 colour scale units of the tissue specific value. 3D mask predictions were obtained by iterating over the 2D segmented slices.

### 2.3 Network specifications

This work uses the “pix2pix” framework of a conditional GAN (cGAN) described by Nvidia (Isola et al., 2017). The cGAN consists of two deep neural networks, a generator (*G*) and a discriminator (*D*). For our task, *G* learns to translate sagittal MR images of the knee joint (source images *x*) to semantic segmentation maps (*G(x)*), while *D* aims to differentiate between the real segmentation map (*y*) and the synthetically generated.

**Fig. 1:**
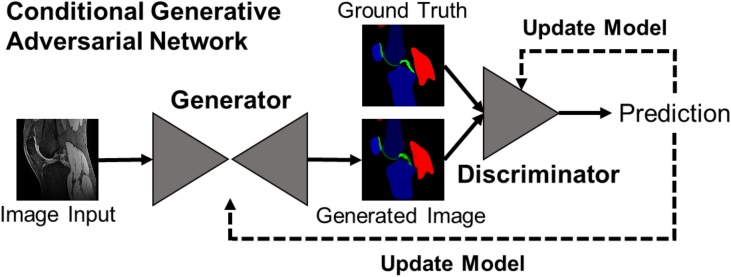
Conditional GAN structure. The generator is a U-Net that progressively down-samples / encodes and then up-samples / decodes an input by a series of convolutional layers, with additional skip-connections between each major layer. The generated, ’fake’ segmentation image is then fed together with the ground truth segmentation image into a discriminator network (PatchGAN (Isola et al., 2017)) that gives its prediction of whether the generated image is a ‘real’ representation of the ground truth image, or not. A detailed description of the network architecture can be found in the Appendix.

The structure of a cGAN is illustrated in Fig. 1. The loss function for this cGAN is

(1) $$L_{cGAN}\left(G,D\right)\;=E_{x,y}\left[\log D\left(x,y\right)\right]+E_{x}[\log(1-D(x,G(x))]$$

The loss function describes how *G* is minimized against a maximised *D*. Since both optimisation processes are dependent on each other, convergence is achieved by reaching a saddle point (simultaneously minimum / maximum for both networks' cost) rather than a minimum. The loss also incorporates a L1 distance to reduce image blurring and ensure that the generated image from *G(x)* are not significantly different from the target image *y* (Isola et al., 2017; Regmi and Borji, 2018). This L1 loss is given by

(2) $$L_{L1}(G)\;=E_{x,y}[||y-G(x)|{|}_{1}]$$

The overall objective of the cGAN is to find the optimal solution to

(3) $$G*\;=\text{arg}\underset{G}{\text{min}}\underset{D}{\text{max}}L_{cGAN}(G,D)\;+\;\lambda L_{L1}(G)$$

with λ being a hyper-parameter used for balancing the two losses (Regmi and Borji, 2018).

The cGAN used in this work utilises the U-Net encoder-decoder architecture for the generator, which is frequently used for image segmentation problems (Ronneberger et al., 2015). The generator was trained to generate images that are indistinguishable from a target image (i.e., the segmented map). Spatial consistency of the data is not guaranteed with a U-Net segmented map, which can cause inaccurate boundaries (Ronneberger et al., 2015). However, adversarial losses in the discriminator regulate and therefore increase the accuracy to higher order shapes (Yang et al., 2017).

We modified the U-Net generator from the “pix2pix” network by increasing the input layer to be able to train on 512 × 512 resolution images. For this an additional Convolution-BatchNorm-leakyReLU layer was inserted in the encoding and a Convolution-BatchNorm-ReLU layer in the decoding network part.

The discriminator is a patch-based fully convolutional neural network, PatchGAN (Li and Wand, 2016; Long et al., 2018), which models the image as a Markov random field. It performs a convolutional patch-wise (N x N) classification with all the outputs in the patch averaged and taken as the output of *D*. *D* is therefore less dependent on distant pixels/voxels beyond a “patch diameter” and is a form of neighbouring texture loss. The PatchGAN can be applied to arbitrarily large images, due to a fixed size of the patch.

To analyse the cGANs performance we compared it to the performance of a U-Net network, which is widely used for image segmentation processes. We used the cGAN generator network as the U-Net network to maintain an effective comparison.

The networks were implemented using PyTorch (Torch v1.0.1) and all training was performed on a Nvidia P6000 GPU card (3840 CUDA cores, 24 GB GDDR5X). The training phase of optimisation was performed as described by the “pix2pix” network, using stochastic gradient descent to minimise D(x,y) and stochastic gradient ascent to maximise D(x,G(x)). The Adam solver was used with a learning rate 0.0002 and momentum parameters,$\beta_{1}=0.5$$\beta_{2}=0.999$. We introduced random noise (jitter) during training by resizing the input images to 542 × 542 using bi-cubic interpolation followed by random cropping back to 512 × 512.

A detailed description of the network architectures can be found in the Appendix.

### 2.4 Segmentation evaluation metrics

The Sørensen–Dice Similarity Coefficient (DSC) (Dice, 1945; Sørensen, 1948) was used to evaluate the overlap between the generated segmentation and the manual segmentation. The DSC ranges between 0 and 1, with 0 representing no overlap and 1 complete overlap between the two sets. DSC is defined as twice the size of the intersect divided by the sum of the sizes of two sample sets, given as

(4) $$DSC=\frac{2|X\cap Y|}{\left|X|+|Y\right|}$$

for Boolean metrics. For the experiments involving the SKI10 and OAI ZIB datasets, the volumetric overlap error (VOE) and the boundary distance-based metric average surfaces distance (ASD) were determined to assess segmentation accuracy and allow an appropriate comparison with previous studies using these datasets. The VOE can be calculated as

(5) $$VOE=1-\;\frac{\left|X\cap Y\right|}{\left|X\cup Y\right|}$$

with small values for VOE expressing greater accuracy.

The ASD is expressed in *mm* and is defined as

(6) $$ASD=\frac{1}{N_{X}+N_{Y}}\left(\sum_{i=1}^{N_{X}}D_{X}(y)+\;\sum_{i=1}^{N_{Y}}D_{Y}(x)\right)$$

where $D_{X}\left(y\right)=\;\underset{x\in X}{\text{min}}\left\|y-x\right\|$ is the distance of a voxel y to a surface X and $\left\|∙\right\|$ denotes the Euclidean norm.

### 2.5 Evaluation of network characteristics

This section aims at evaluating and adjusting specific network characteristics towards improving overall network performance, for both cGAN and U-Net. All networks in this section were trained for 100 epochs and all cGANs with a 70 × 70 PatchGAN discriminator unless otherwise stated.

#### 2.5.1 Evaluation of network objective function

We evaluated the cGANs performance with different objective functions by combining the cGAN loss with different pixel-wise error losses. In this work the cGAN is tasked to output a segmentation map of multiple tissues having different features and locations in the input MR image. We assessed the shortcomings and strengths of including the $L_{L1}$, $L_{L2}$ and Smooth L1 ($L_{SmL1}$) (Girshick, 2015) loss functions in the cGAN objective. The $L_{L2}$ loss and $L_{SmL1}$ loss are given by

(7) $$L_{L2}(G)\;=E_{x,y}[||y-G(x)|{|}_{2}^{2}]$$

(8) LSmL1G =0.5∙Ex,y[||y-G(x)||22] , if |y-G(x)| < 1  Ex,y[||y-G(x)||1]-0.5 , otherwise  

Furthermore, the weighting hyperparameter λ between the cGAN loss and pixel-wise error loss was changed to vary the balance between the two task losses. λ = 0.01, 1, 100 and 10,000 were investigated. Network training with the cGAN loss alone (λ = 0) was additionally performed and evaluated.

We also trained the U-Net with the same three different pixel-wise error losses ($L_{L1}$, $L_{L2}$ and $L_{SmL1}$) as the cGAN to maintain an effective comparison.

#### 2.5.2 Evaluation of altering the loss objective during training

After obtaining initial results, we observed that the cGAN was unable to segment muscle tissues, independent of the objective function trained on. Therefore, we decided to explore the effect of varying the loss objective during training. For this, we trained a cGAN with $L_{cGAN}+\lambda L_{L2}$ loss and a U-Net with $L_{L2}$ loss for 50 epochs and then changed the loss functions for the ensuing 50 epochs to $L_{cGAN}+\lambda L_{L1}$ and $L_{L1}$, respectively.

#### 2.5.3 Evaluation of the generator depth

We analysed the effect of changing the depth of the generator network on the cGANs and U-Nets quantitative performance. In addition to the generator down-sampling the input through nine convolutional networks, we tested a generator consisting of seven and five convolutions during down-sampling. Furthermore, we assessed the quantitative performance of the generator network with different numbers feature channels. We compared networks starting with different minimum number of feature channels (16, 32, 64 and 128) and thus end at different maximum numbers of feature channels (128, 256, 512 and 1024). All cGANs were trained with $L_{cGAN}+\lambda L_{L1}$ loss with λ = 100 and all U-Nets with the $L_{L1}$ loss. Detailed descriptions of the generator network architectures can be found in the Appendix.

#### 2.5.4 Evaluation of the PatchGAN receptive field size

We evaluated the effect of changing the PatchGAN receptive field size on the cGANs qualitative (artefact emergence) and quantitative (segmentation accuracy) performance. In addition to the 70 × 70 PatchGAN, we tested a 1 × 1 (PixelGAN), 34 × 34 and 286 × 286 PatchGAN. All cGANs were trained with $L_{cGAN}+\lambda L_{L1}$ loss with λ = 100. Detailed descriptions of the discriminator network architectures can be found in the Appendix.

#### 2.5.5 Evaluation of transfer learning

Since the AMROA dataset only comprises of a low number of subjects (N = 8) for training, we assess the influence of transfer learning on network performance, by initially training both a cGAN ($L_{cGAN}+\lambda L_{L1}$) and a U-Net ($L_{L1}$) for 20 epochs on the larger SKI10 and OAI ZIB training datasets separately followed by network fine-tuning for 80 epochs on the smaller AMROA training set. Additionally, a cGAN and a U-Net were trained for 20 epochs on the AMROA training dataset followed by network refinement training for 80 epochs on either the SKI10 or OAI ZIB training set to analyse the potential segmentation improvement of SKI10 and OAI ZIB. Network performance evaluations were performed using AMROA, SKI10 and OAI ZIB testing datasets. As determined from the previous sections, the cGAN trained with the $L_{cGAN}+\lambda L_{L1}$ loss objective (λ = 100) and a 1 × 1 PixelGAN as well as the U-Net trained with the $L_{L1}$ loss objective achieved the highest segmentation accuracies for most knee joint tissues segmented in the AMROA dataset and were used in this section.

## 3 Results and discussion

### 3.1 Network training and testing

Semi-manual segmentation of the AMROA images by the reader required −30 min per subject-volume. Segmentation post-training on a single slice was processed in ≈0.13 s. A detailed description of all cGAN and U-Net training durations for all datasets can be found in the Appendix. The highlights of the upcoming sections are:

**Table:** 

3.2	The U-Net trained with $L_{L1}$ loss objective outperformed the cGANs and the U-Nets trained with different loss objectives in the segmentation performance of most knee joint tissues.
3.3	Altering the network objective function midway through cGAN and U-Net training lead to unanticipated but advantageous results. This variation resulted in improved segmentation performances of several tissues and the cGANs capability to segment muscle tissue, which previously had not been possible with non-altered objective function training.
3.4	The cGAN and U-Net trained with nine convolutions/transpose convolutions in the networks encoding/decoding parts and a minimum feature channel change of 64 achieved the highest segmentation accuracies for most knee joint tissues annotated.
3.5	The greatest improvements in segmentation performance of the cGAN was achieved by reducing the receptive field size of the discriminator network. This resulted in segmentation accuracies equivalent to those of the U-Net.
3.6	Transfer learning not only increased segmentation accuracy of some tissues of the fine-tuned dataset, but also increased the network’s capacity to maintain segmentation capabilities for the pretrained dataset.
3.7	Overall, the cGAN trained with the $L_{cGAN}+\lambda L_{L1}$ loss objective (λ = 100) and a 1 × 1 PixelGAN as well as the U-Net trained with the $L_{L1}$ loss objective achieved comparable and the highest segmentation accuracies for most knee joint tissues segmented.

### 3.2 Evaluation of network objective function

**Fig. 2:**
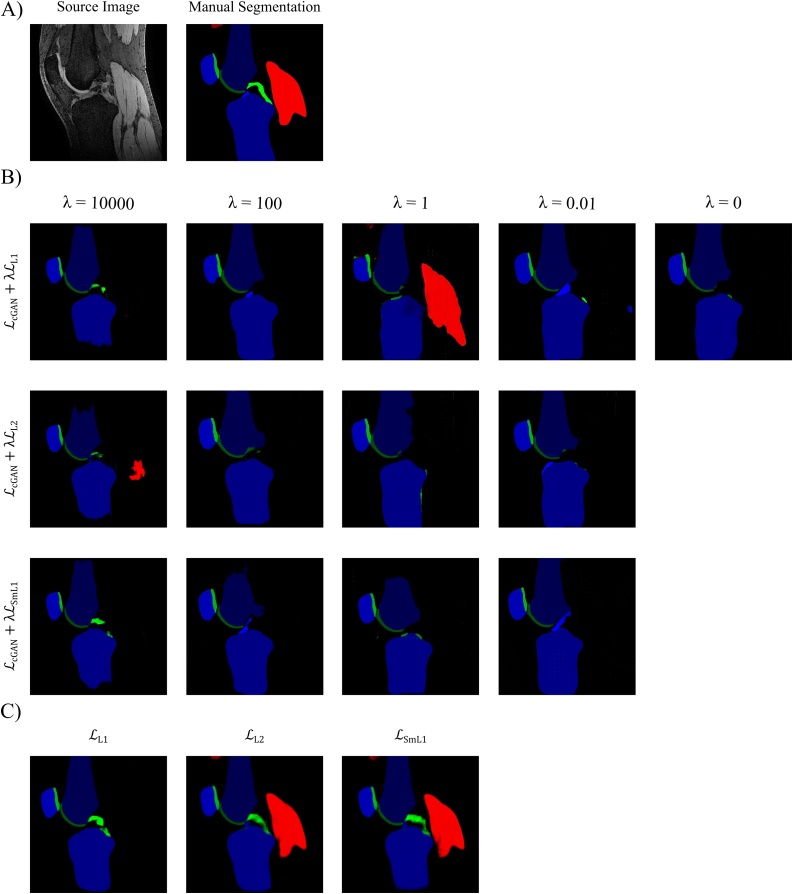
Results of Network Objective Function. Qualitative results of B) training a cGAN with different objective functions by combining the cGAN loss with different pixel-wise error losses with varying weightings and C) training a U-Net with different pixel-wise error losses.

The quantitative results of assessing the impact of combining the cGAN objective with three different pixel error losses with varying weightings λ on the cGANs segmentation performance are in Table 2, with the qualitative results depicted in Fig. 2B. The cGANs trained with larger values for λ (λ = 100 and 10,000) achieved the highest segmentation performance for all tissues and the produced segmentation maps were less affected by artefacts compared to the cGANs trained with λ = 0.01 and 1. For instance, the images from the networks trained with $L_{cGAN}+\lambda L_{L1}$ (λ = 0.01), $L_{cGAN}+\lambda L_{L2}$ (λ = 1) and $L_{cGAN}+\lambda L_{SmL1}$ (λ = 1) had artefacts where the networks seem to detect bone or cartilage structures where there were none in the original MR input image. By increasing the weighting hyperparameter λ, more emphasis is put on the pixel error losses to guide the network to produce more accurate representations of the ground truth segmentation map and reduces these artefacts. However, the influence of GAN loss diminishes with very large values for λ with the discriminator having minimal effect on generator training.

**Table 2:** Results of the Network Objective Function: cGAN. The influence of mixing the cGAN objective with different pixel-wise error losses and varying their significance by changing the weighting hyperparameter λ on the segmentation performance of the proposed cGAN was assessed. Highest DSCs achieved for each tissue are in bold.

Network Objective Function Results											
cGAN											
Pixel Loss	*λ*	F Bone	T Bone	P Bone	F Cartilage	T Cartilage	P Cartilage	VM Muscle	GM Muscle	ACL	PCL
L1	*0*	0.931 ± 0.020	0.864 ± 0.008	0.911 ± 0.036	0.774 ± 0.030	0.717 ± 0.108	0.872 ± 0.030	0.000 ± 0.000	0.000 ± 0.000	0.000 ± 0.000	0.000 ± 0.000
	*0.01*	0.900 ± 0.018	0.890 ± 0.031	0.912 ± 0.002	0.727 ± 0.023	0.715 ± 0.060	0.850 ± 0.048	0.000 ± 0.000	0.000 ± 0.000	0.509 ± 0.009	0.171 ± 0.208
	*1*	0.899 ± 0.014	0.856 ± 0.010	0.807 ± 0.060	0.465 ± 0.037	0.666 ± 0.022	0.426 ± 0.098	0.611 ± 0.181	0.595 ± 0.054	0.000 ± 0.000	0.000 ± 0.000
	*100*	0.918 ± 0.011	0.948 ± 0.018	0.928 ± 0.002	0.812 ± 0.002	0.748 ± 0.042	**0.863 ± 0.043**	0.113 ± 0.085	0.000 ± 0.000	0.577 ± 0.020	0.073 ± 0.103
	10,000	**0.968 ± 0.006**	0.944 ± 0.026	0.917 ± 0.008	**0.875 ± 0.021**	**0.810 ± 0.036**	0.840 ± 0.065	0.879 ± 0.036	0.793 ± 0.080	0.432 ± 0.237	0.338 ± 0.386
L2	*0.01*	0.902 ± 0.004	0.915 ± 0.003	0.923 ± 0.005	0.750 ± 0.002	0.740 ± 0.079	0.834 ± 0.077	0.000 ± 0.000	0.000 ± 0.000	0.000 ± 0.000	0.000 ± 0.000
	*1*	0.902 ± 0.046	0.902 ± 0.008	0.902 ± 0.044	0.741 ± 0.004	0.736 ± 0.033	0.838 ± 0.041	0.000 ± 0.000	0.000 ± 0.000	0.149 ± 0.104	0.002 ± 0.002
	*100*	0.928 ± 0.015	0.939 ± 0.007	0.921 ± 0.022	0.768 ± 0.016	0.752 ± 0.049	0.862 ± 0.039	0.001 ± 0.001	0.000 ± 0.000	**0.652 ± 0.094**	0.101 ± 0.074
	10,000	0.952 ± 0.000	**0.950 ± 0.015**	0.923 ± 0.001	0.828 ± 0.043	0.684 ± 0.092	0.832 ± 0.054	0.814 ± 0.145	0.856 ± 0.121	0.440 ± 0.084	0.293 ± 0.358
SmL1	*0.01*	0.914 ± 0.034	0.902 ± 0.003	0.920 ± 0.011	0.726 ± 0.007	0.729 ± 0.042	0.762 ± 0.068	0.000 ± 0.000	0.000 ± 0.000	0.343 ± 0.066	0.000 ± 0.000
	*1*	0.884 ± 0.044	0.912 ± 0.006	0.926 ± 0.013	0.740 ± 0.014	0.732 ± 0.044	0.829 ± 0.067	0.055 ± 0.007	0.000 ± 0.000	0.000 ± 0.000	0.000 ± 0.000
	*100*	0.903 ± 0.019	0.944 ± 0.006	**0.936 ± 0.003**	0.776 ± 0.035	0.741 ± 0.066	0.857 ± 0.029	0.031 ± 0.044	0.070 ± 0.100	0.578 ± 0.053	0.044 ± 0.052
	10,000	0.951 ± 0.002	0.946 ± 0.018	0.935 ± 0.015	0.825 ± 0.035	0.738 ± 0.047	0.797 ± 0.088	**0.914 ± 0.001**	**0.837 ± 0.146**	0.261 ± 0.073	**0.374 ± 0.341**

Training and testing were performed on the AMROA training and testing datasets, respectively.

DSCs presented as mean ± standard deviation.

Abbreviations: F Bone – femoral bone, T Bone – tibial bone, P Bone – patellar bone, F Cartilage – femoral cartilage, T Cartilage – tibial cartilage, P Cartilage – patellar cartilage, VM Muscle - vastus medialis muscle, GM Muscle – medial head of gastrocnemius medialis muscle, ACL – anterior cruciate ligament, PCL – posterior cruciate ligament, DSC - Sørensen–Dice similarity coefficient.

The qualitative results of training a U-Net with different pixel error losses are presented in Fig. 2C while the quantitative results are listed in Table 3. The U-Net trained with $L_{L1}$ loss objective achieves the highest accuracy for all tissues compared to $L_{L2}$ and $L_{SmL1}$ loss except for the muscle tissues. Muscle tissues appeared on the majority of 2D MR knee images seen by the network during training, however we only segmented two selective medial muscles in the AMROA dataset due to time constraints. It is interesting to note that although the U-Net trained with $L_{L1}$ was not able to capture the medial head of gastrocnemius and vastus medialis muscles, the cGAN trained with the $L_{cGAN}+\lambda L_{L1}$ objective (λ = 10,000) was. Simple absolute difference ($L_{L1}$) was not capable of differentiating lateral muscle textures from medial. The U-Nets trained with $L_{L2}$ and $L_{SmL1}$ losses were capable of segmenting the selective muscles with high accuracies as they are penalised more by the squaring term in their loss objectives when the difference between ground truth and model predictions are large. Interestingly, although the patella bone and cartilage only appear on very few slices in a 3D dataset, and ACL and PCL on even fewer, the U-Net with $L_{L1}$ segmented these tissues better than the $L_{L2}$ and $L_{SmL1}$ ($L_{L2}$: DSC_P Bone_ < 0.2 %, DSC_P Cartilage_ < 5.3 %, DSC_ACL_ < 15.2 %, DSC_PCL_ < 21.3 %; $L_{SmL1}$ : DSC_P Bone_ < 0.4 %, DSC_P Cartilage_ < 6.0 %, DSC_ACL_ < 6.9 %, DSC_PCL_ < 17.8 %). This could be explained by the cruciate ligament and patellar tissues either being present or not on a 2D training image and the network is not being constrained to only segment medial tissues. Overall, the U-Net with $L_{L1}$ produced sharper boundaries, especially for the smaller ligament structures, as compared to the segmentation maps produced by U-Nets trained with $L_{L2}$ and $L_{SmL1\;}$, in which the boundaries are more diffused.

**Table 3:** Results of the Network Objective Function: U-Net. The influence of different pixel-wise error losses on the segmentation performance of the U-Net was assessed. Highest DSCs achieved for each tissue are in bold.

Network Objective Function Results										
U-Net										
Pixel Loss	F Bone	T Bone	P Bone	F Cartilage	T Cartilage	P Cartilage	VM Muscle	GM Muscle	ACL	PCL
L1	**0.972 ± 0.006**	**0.960 ± 0.001**	**0.941 ± 0.010**	**0.886 ± 0.007**	**0.834 ± 0.010**	**0.890 ± 0.034**	0.000 ± 0.000	0.000 ± 0.000	**0.643 ± 0.153**	**0.641 ± 0.008**
L2	0.950 ± 0.007	0.957 ± 0.009	0.939 ± 0.003	0.831 ± 0.020	0.723 ± 0.068	0.837 ± 0.051	0.888 ± 0.000	0.881 ± 0.021	0.491 ± 0.136	0.428 ± 0.196
SmL1	0.953 ± 0.001	0.953 ± 0.009	0.937 ± 0.004	0.843 ± 0.021	0.771 ± 0.036	0.830 ± 0.088	**0.894 ± 0.002**	**0.910 ± 0.045**	0.574 ± 0.230	0.463 ± 0.174

Training and testing were performed on the AMROA training and testing datasets, respectively.

DSCs are presented as mean ± standard deviation.

Abbreviations: F Bone – femoral bone, T Bone – tibial bone, P Bone – patellar bone, F Cartilage – femoral cartilage, T Cartilage – tibial cartilage, P Cartilage – patellar cartilage, VM Muscle - vastus medialis muscle, GM Muscle – medial head of gastrocnemius medialis muscle, ACL – anterior cruciate ligament, PCL – posterior cruciate ligament, DSC - Sørensen–Dice similarity coefficient.

**Fig. 3:**
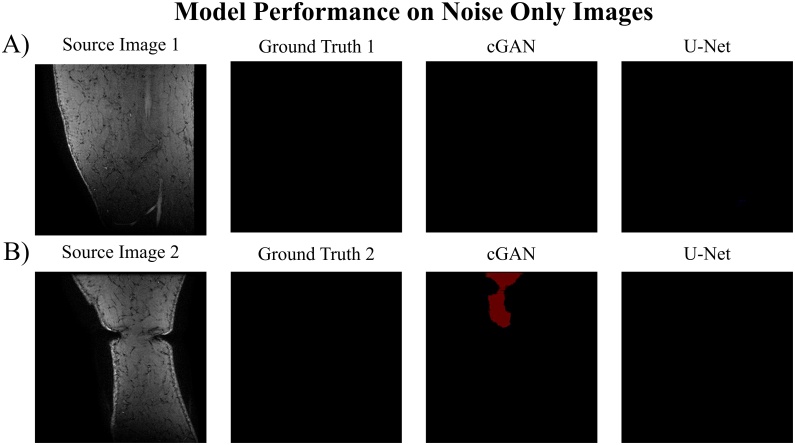
Results of testing on noise only images. Assessing the segmentation performance of a cGAN trained with $L_{\mathrm{cGAN}}+\lambda L_{L1}\;(\lambda =100)$ loss objective and a U-Net trained with $L_{L1}$ objective and tested on noise only images. Training was performed on the AMROA training dataset without noise only images. A) and B) are two example results of testing the models on noise only source images and comparing to ground truth segmentation maps.

We decided to assess the model’s performance when including noise-only images in the testing dataset as we excluded them during model training, and this might limit the models’ use in a clinical setting. This effect was only evaluated for a the cGAN trained with the $L_{cGAN}+\lambda L_{L1}$ (λ = 100) objective function and the U-Net trained with the $L_{L1}$ loss objective. The quantitative results are listed in Table 4 with qualitative results displayed in Fig. 3. Both networks showed comparable segmentation performances after testing with noise-only images with percentage differences (%-Diff) of the DSC for all segmented tissues ≤ 2.3 %. Including noise-only images into the testing set had greater effects on the cGAN DSC of the medial vastus muscle (VM muscle) (%-Diff = 1.5 %), the ACL (%-Diff = 1.6 %) and the PCL (%-Diff = 1.9 %) as well as on the U-Net DSC of the ACL (%-Diff = 2.3 %). These higher differences could be explained by the lower segmentation capability of these structures by the cGAN and U-Net models to begin with (cGAN: DSC_VM muscle_: 0.113 vs 0.098, DSC_ACL_: 0.577 vs 0.593; DSC_PCL_: 0.073 vs 0.092; U-Net: DSC_ACL_: 0.643 vs 0.620). Furthermore, the larger %-Diff in the DSC of the VM muscle is caused by the cGAN model irregularly segmenting VM muscle tissues on noise only images (Fig. 3B).

**Table 4:** Results of additionally testing on noise only images. The influence of including noise only images in the testing set on the overall segmentation performance of a cGAN trained with $L_{\mathrm{cGAN}}+\lambda L_{L1}\;(\lambda =100)$ loss objective and a U-Net trained with $L_{L1}$ objective. Training was performed on the AMROA training dataset without noise only images.

Influence of Noise Only Images										
cGAN										
Testing	F Bone	T Bone	P Bone	F Cartilage	T Cartilage	P Cartilage	VM Muscle	GM Muscle	ACL	PCL
No Noise	0.918 ± 0.011	0.948 ± 0.018	0.928 ± 0.002	0.812 ± 0.002	0.748 ± 0.042	0.863 ± 0.043	0.113 ± 0.085	0.000 ± 0.000	0.577 ± 0.020	0.073 ± 0.103
With Noise	0.925 ± 0.012	0.946 ± 0.017	0.928 ± 0.004	0.810 ± 0.003	0.752 ± 0.045	0.858 ± 0.054	0.098 ± 0.114	0.000 ± 0.000	0.593 ± 0.028	0.092 ± 0.131
%-Diff	0.7	0.2	0.0	0.2	0.4	0.5	1.5	0.0	1.6	1.9

U-Net										
Testing	F Bone	T Bone	P Bone	F Cartilage	T Cartilage	P Cartilage	VM Muscle	GM Muscle	ACL	PCL
No Noise	0.972 ± 0.006	0.960 ± 0.001	0.941 ± 0.010	0.886 ± 0.007	0.834 ± 0.010	0.890 ± 0.034	0.000 ± 0.000	0.000 ± 0.000	0.643 ± 0.153	0.641 ± 0.008
With Noise	0.968 ± 0.001	0.957 ± 0.009	0.938 ± 0.016	0.885 ± 0.004	0.833 ± 0.010	0.894 ± 0.026	0.000 ± 0.000	0.000 ± 0.000	0.620 ± 0.156	0.643 ± 0.025
%-Diff	0.4	0.3	0.3	0.1	0.1	0.4	0.0	0.0	2.3	0.2

DSCs are presented as mean ± standard deviation.

Abbreviations: F Bone – femoral bone, T Bone – tibial bone, P Bone – patellar bone, F Cartilage – femoral cartilage, T Cartilage – tibial cartilage, P Cartilage – patellar cartilage, VM Muscle - vastus medialis muscle, GM Muscle – medial head of gastrocnemius medialis muscle, ACL – anterior cruciate ligament, PCL – posterior cruciate ligament, DSC - Sørensen–Dice similarity coefficient, %-Diff – absolute percentage difference.

### 3.3 Evaluation of altering loss objective during training

**Fig. 4:**
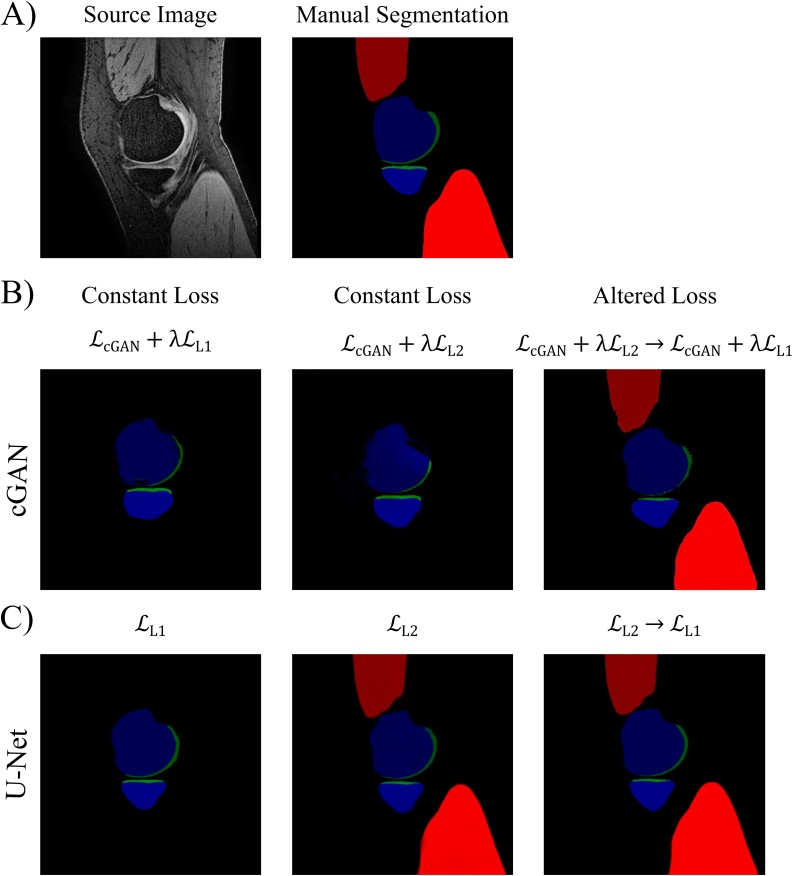
Results of Altering the Loss Objective during Training. Assessing the influence of varying the objective function halfway during cGAN and U-Net training on their segmentation performance with comparison to the respective cGANs and U-Nets trained with constant loss function.

**Fig. 5:**
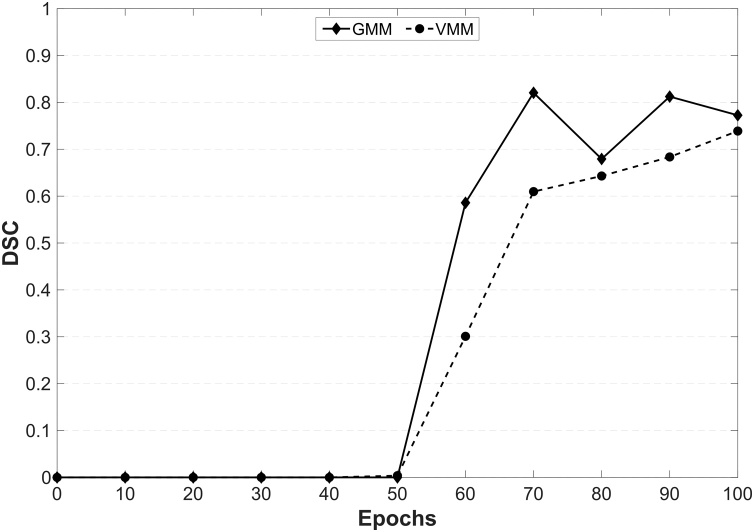
Influence of altering the loss objective during cGAN training on the segmentation performance of the medial gastrocnemius and vastus muscles. The cGAN was trained with a $L_{\text{c}\text{G}\text{A}\text{N}}+\text{λ}L_{\text{L}2}$ loss objective for 50 epochs followed by a further 50 epochs training with $L_{\text{c}\text{G}\text{A}\text{N}}+\text{λ}L_{\text{L}1}$. Abbreviations: VMM - vastus medialis muscle, GMM – medial head of gastrocnemius muscle, DSC – Dice Similarity Coefficient

Fig. 4 compares the qualitative results and Table 5 compares the DSCs obtained from a cGAN and a U-Net, in which the objective functions were changed midway through training to the cGANs and U-Nets trained with non-altered objective functions. Training a cGAN with varied loss objective ($L_{cGAN}+\lambda L_{L2}$ → $L_{cGAN}+\lambda L_{L1}$) notably reduced its ability to segment the ACL, however considerably improved its segmentation performance on the medial vastus and gastrocnemius muscles, as well as PCL, compared to the other cGANs ($L_{cGAN}+\lambda L_{L1}$ and $L_{cGAN}+\lambda L_{L2}$). The images in Fig. 4B show the improvements in muscle segmentation with the cGAN trained with varied loss objective. This was a surprising result as neither the cGAN trained with $L_{cGAN}+\lambda L_{L1}$ nor with $L_{cGAN}+\lambda L_{L2}$ alone were able to segment muscle. Looking at the different training epochs of the cGAN trained with varied loss, during $L_{cGAN}+\lambda L_{L2}$ no muscle tissue was being semantically segmented. However, when changing to $L_{cGAN}+\lambda L_{L1}$ and between training epochs 50 and 60, the network started segmenting muscle tissue (Fig. 5). After the initial 50 epochs of $L_{cGAN}+\lambda L_{L2}$ training, the cGANs weights must have been favourable for continuing training with $L_{cGAN}+\lambda L_{L1}$ to additionally semantically segment muscle tissue.

**Table 5:** Results of Altering the Loss Objective during Training. Assessing the influence of altering the loss objective function during training on the segmentation performance of the proposed cGAN and U-Net. A cGAN was trained with $L_{\mathrm{cGAN}}+\lambda L_{L2}$ objective and a U-Net with $L_{L2}$ objective for 50 epochs followed by a further 50 epochs training with $L_{\mathrm{cGAN}}+\lambda L_{L1}$ and $L_{L1}$ objectives, respectively. Segmentation performances are compared with the previously trained cGANs ($L_{\mathrm{cGAN}}+\lambda L_{L1}\;\mathrm{and}\;L_{\mathrm{cGAN}}+\lambda L_{L2}$; λ=100; 100 epochs) and U-Nets ($L_{L1}\;\mathrm{and}\;L_{L2}$;100 epochs). Highest DSCs achieved for each tissue are in bold.

Altering the Loss Objective during Training Results										
cGAN										
Network Loss Objective	F Bone	T Bone	P Bone	F Cartilage	T Cartilage	P Cartilage	VM Muscle	GM Muscle	ACL	PCL
$L_{cGAN}+\lambda L_{L1}$	0.918 ± 0.011	**0.948 ± 0.018**	**0.928 ± 0.002**	**0.812 ± 0.002**	0.748 ± 0.042	**0.863 ± 0.043**	0.113 ± 0.085	0.000 ± 0.000	0.577 ± 0.020	0.073 ± 0.103
$L_{cGAN}+\lambda L_{L2}$	0.928 ± 0.015	0.939 ± 0.007	0.921 ± 0.022	0.768 ± 0.016	0.752 ± 0.049	0.862 ± 0.039	0.001 ± 0.001	0.000 ± 0.000	**0.652 ± 0.094**	0.101 ± 0.074
$L_{cGAN}+\lambda L_{L2}$ → $L_{cGAN}+\lambda L_{L1}$	**0.936 ± 0.007**	0.938 ± 0.021	0.884 ± 0.078	0.800 ± 0.021	**0.760 ± 0.035**	0.855 ± 0.031	**0.739 ± 0.010**	**0.772 ± 0.005**	0.115 ± 0.032	**0.392 ± 0.128**

U-Net										
Network Loss Objective	F Bone	T Bone	P Bone	F Cartilage	T Cartilage	P Cartilage	VM Muscle	GM Muscle	ACL	PCL
$L_{L1}$	**0.972 ± 0.006**	0.960 ± 0.001	0.941 ± 0.010	**0.886 ± 0.007**	**0.834 ± 0.010**	**0.890 ± 0.034**	0.000 ± 0.000	0.000 ± 0.000	**0.643 ± 0.153**	**0.641 ± 0.008**
$L_{L2}$	0.950 ± 0.007	0.957 ± 0.009	0.939 ± 0.003	0.831 ± 0.020	0.723 ± 0.068	0.837 ± 0.051	0.888 ± 0.000	0.881 ± 0.021	0.491 ± 0.136	0.428 ± 0.196
$L_{L2}$ → $L_{L1}$	0.970 ± 0.006	**0.961 ± 0.007**	**0.941 ± 0.003**	0.869 ± 0.016	0.793 ± 0.021	0.886 ± 0.027	**0.914 ± 0.008**	**0.933 ± 0.010**	0.632 ± 0.170	0.567 ± 0.094

Training and testing were performed on the AMROA training and testing datasets, respectively.

DSCs are presented as mean ± standard deviation.

Abbreviations: FB – femoral bone, TB – tibial bone, PB – patellar bone, FC – femoral cartilage, TC – tibial cartilage, PC – patellar cartilage, VM Muscle - vastus medialis muscle, GM Muscle – medial head of gastrocnemius medialis muscle, ACL – anterior cruciate ligament, PCL – posterior cruciate ligament, DSC - Sørensen–Dice similarity coefficient.

The U-Net trained with altered objective function ($L_{L2}$ → $L_{L1}$) also showed notable improvements in the segmentation performance of the medial vastus and gastrocnemius muscles while the segmentation scores of the other knee tissues remained comparable with those of the other U-Nets ($L_{L1}$ and $L_{L2}$). Fig. 4C qualitatively compares the results of a U-Net trained with altered loss objective to those of the U-Nets trained with a single, non-altered loss objective. As mentioned in the corresponding method section, this idea came after reviewing a few initial training results. While the U-Net trained with the $L_{L1}$ objective was not able to segment the medial vastus and gastrocnemius muscles after training, the U-Net with the $L_{L2}$ loss objective was. However, these images were slightly blurrier, and the segmentation accuracy of the remaining tissues was poorer than compared to $L_{L1}$. By varying the loss objective during training, the strengths of $L_{L2}$ and $L_{L1}$ were combined. We decided to first train the network with $L_{L2}$ loss to capture all tissues and then to change to $L_{L1}$ halfway through training to make the images sharper and increase segmentation accuracy. This method created a more proficient network capable of segmenting all tissues with higher or comparable accuracies to the networks trained with non-altered loss objectives.

### 3.4 Evaluation of the generator depth

The quantitative results of assessing the impact of generator network depth on the cGANs and U-Nets segmentation performances are in Table 6, Table 7.

**Table 6:** Results of Varying Generator Network Depth: Number of Convolutions. The influence of varying the number of convolutions during down-sampling in the generator networks of both the cGAN and U-Net was assessed. Highest DSCs achieved for each tissue are in bold.

Generator Network Depth Results – Number of Convolutions during Down-Sampling										
cGAN										
Down Convs	F Bone	T Bone	P Bone	F Cartilage	T Cartilage	P Cartilage	VM Muscle	GM Muscle	ACL	PCL
5	**0.928 ± 0.006**	0.929 ± 0.006	0.893 ± 0.029	0.721 ± 0.029	**0.751 ± 0.039**	0.838 ± 0.042	0.049 ± 0.069	0.000 ± 0.000	0.622 ± 0.042	0.286 ± 0.189
7	0.889 ± 0.023	0.921 ± 0.026	0.928 ± 0.002	0.764 ± 0.047	0.624 ± 0.039	0.846 ± 0.057	**0.171 ± 0.226**	**0.167 ± 0.236**	**0.626 ± 0.041**	**0.289 ± 0.408**
9	0.918 ± 0.011	**0.948 ± 0.018**	**0.928 ± 0.002**	**0.812 ± 0.002**	0.748 ± 0.042	**0.863 ± 0.043**	0.113 ± 0.085	0.000 ± 0.000	0.577 ± 0.020	0.073 ± 0.103

U-Net										
Down Convs	F Bone	T Bone	P Bone	F Cartilage	T Cartilage	P Cartilage	VM Muscle	GM Muscle	ACL	PCL
5	0.969 ± 0.002	0.952 ± 0.016	0.919 ± 0.022	**0.887 ± 0.018**	0.823 ± 0.001	0.888 ± 0.031	0.000 ± 0.000	0.000 ± 0.000	0.631 ± 0.125	0.544 ± 0.249
7	0.964 ± 0.003	0.956 ± 0.005	0.921 ± 0.008	0.874 ± 0.032	0.787 ± 0.044	0.869 ± 0.029	0.000 ± 0.000	0.000 ± 0.000	0.539 ± 0.160	0.592 ± 0.120
9	**0.972 ± 0.006**	**0.960 ± 0.001**	**0.941 ± 0.010**	0.886 ± 0.007	**0.834 ± 0.010**	**0.890 ± 0.034**	0.000 ± 0.000	0.000 ± 0.000	**0.643 ± 0.153**	**0.641 ± 0.008**

Training and testing were performed on the AMROA training and testing datasets, respectively.

DSCs are presented as mean ± standard deviation.

Abbreviations: F Bone – femoral bone, T Bone – tibial bone, P Bone – patellar bone, F Cartilage – femoral cartilage, T Cartilage – tibial cartilage, P Cartilage – patellar cartilage, VM Muscle - vastus medialis muscle, GM Muscle – medial head of gastrocnemius medialis muscle, ACL – anterior cruciate ligament, PCL – posterior cruciate ligament, DSC - Sørensen–Dice similarity coefficient.

**Table 7:** Results of Varying Generator Network Depth: Number of Minimum Feature Maps. The influence of starting with different numbers of minimum feature channel maps in the generator networks of both the cGAN and U-Net was assessed. Highest DSCs achieved for each tissue are highlighted grey and in bold.

Generator Network Depth Results – Number of Minimum Feature Channel Maps										
cGAN										
Feature Maps	F Bone	T Bone	P Bone	F Cartilage	T Cartilage	P Cartilage	VM Muscle	GM Muscle	ACL	PCL
16	0.774 ± 0.059	0.903 ± 0.040	0.858 ± 0.003	0.547 ± 0.236	0.473 ± 0.269	0.771 ± 0.070	0.000 ± 0.000	0.000 ± 0.000	0.000 ± 0.000	0.000 ± 0.000
32	0.899 ± 0.004	0.937 ± 0.001	0.875 ± 0.027	0.750 ± 0.028	0.720 ± 0.038	0.831 ± 0.030	**0.414 ± 0.260**	0.000 ± 0.000	0.000 ± 0.000	0.000 ± 0.000
64	0.918 ± 0.011	**0.948 ± 0.018**	**0.928 ± 0.002**	**0.812 ± 0.002**	0.748 ± 0.042	**0.863 ± 0.043**	0.113 ± 0.085	0.000 ± 0.000	**0.577 ± 0.020**	**0.073 ± 0.103**
128	**0.925 ± 0.006**	0.935 ± 0.021	0.831 ± 0.032	0.805 ± 0.010	**0.773 ± 0.081**	0.784 ± 0.061	0.341 ± 0.256	0.000 ± 0.000	0.336 ± 0.219	0.011 ± 0.016

U-Net										
Feature Maps	F Bone	T Bone	P Bone	F Cartilage	T Cartilage	P Cartilage	VM Muscle	GM Muscle	ACL	PCL
16	0.966 ± 0.000	0.950 ± 0.021	0.912 ± 0.028	0.868 ± 0.011	0.795 ± 0.001	0.864 ± 0.028	0.000 ± 0.000	0.000 ± 0.000	0.000 ± 0.000	0.202 ± 0.110
32	0.969 ± 0.006	0.946 ± 0.016	0.914 ± 0.005	0.875 ± 0.026	0.795 ± 0.051	0.878 ± 0.032	0.000 ± 0.000	0.000 ± 0.000	0.000 ± 0.000	0.453 ± 0.039
64	**0.972 ± 0.006**	**0.960 ± 0.001**	**0.941 ± 0.010**	**0.886 ± 0.007**	**0.834 ± 0.010**	0.890 ± 0.034	0.000 ± 0.000	0.000 ± 0.000	0.643 ± 0.153	**0.641 ± 0.008**
128	0.968 ± 0.006	0.960 ± 0.004	0.929 ± 0.014	0.884 ± 0.022	0.823 ± 0.010	**0.897 ± 0.013**	0.000 ± 0.000	0.000 ± 0.000	**0.645 ± 0.053**	0.597 ± 0.025

Training and testing were performed on the AMROA training and testing datasets, respectively.

DSCs are presented as mean ± standard deviation.

Abbreviations: F Bone – femoral bone, T Bone – tibial bone, P Bone – patellar bone, F Cartilage – femoral cartilage, T Cartilage – tibial cartilage, P Cartilage – patellar cartilage, VM Muscle - vastus medialis muscle, GM Muscle – medial head of gastrocnemius medialis muscle, ACL – anterior cruciate ligament, PCL – posterior cruciate ligament, DSC - Sørensen–Dice similarity coefficient.

The cGAN with a generator down-sampling the input through nine convolutional networks achieved the highest DSC scores for tibial and patellar bone, as well as for femoral and patellar cartilage. Femoral bone and tibial cartilage were best segmented by the cGAN with five convolutions/transpose convolutions in the generator encoding/decoding parts. The medial vastus and gastrocnemius muscles, as well as ACL and PCL were best segmented by the cGAN with seven convolutions. Training the cGAN with a minimum feature channel change of 64 resulted in the highest segmentation scores for most tissues except for femoral bone, tibial cartilage and the medial vastus muscle.

The U-Net trained with nine convolutions/transpose convolutions in the networks encoding/decoding parts achieved the highest segmentation accuracies for all but one tissue (femoral cartilage), which was slightly better segmented by the U-Net with five convolutions/transpose convolutions. Training the U-Net with a minimum feature channel change of 64 resulted in the highest DSC scores for most tissues apart from patella cartilage and ACL which were segmented best by the U-Net trained with a minimum feature channel change of 128.

It is important to note for this section that increasing the number of convolutions and feature channels in the generator network substantially increases the overall number of parameters in the network and the time per epoch required to train the network (see network architectures in the Appendix for details). A considered decision between increase in learning time and significant improvement in segmentation accuracy has to be made.

### 3.5 Evaluation of PatchGAN receptive field size

**Fig. 6:**
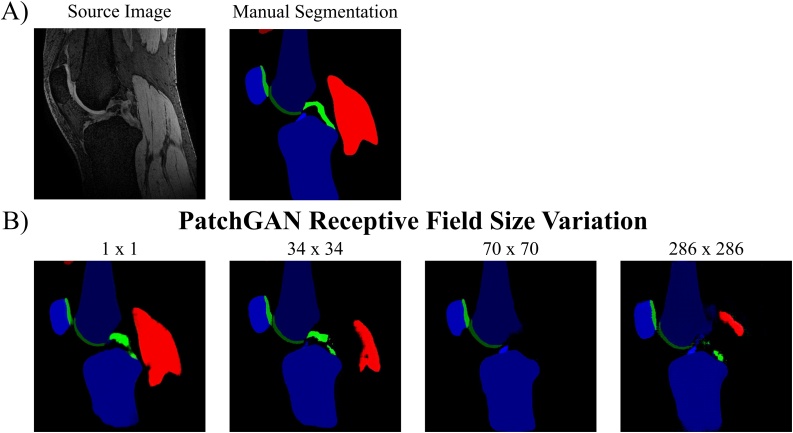
Results of PatchGAN Receptive Field Size. Assessing the influence of varying the discriminator receptive field size on segmentation performance of cGAN when trained and tested on the AMROA dataset.

**Fig. 7:**
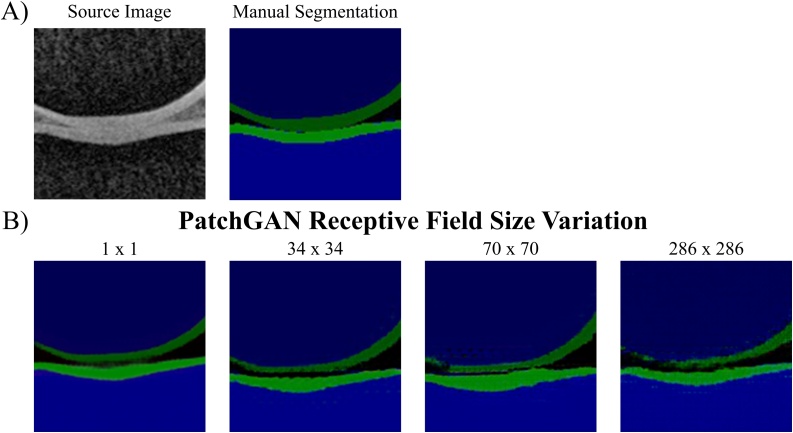
Image Artefact due to the choice of PatchGAN Receptive Field Size. Influence of discriminator receptive field size on checkerboard artefact emergence of a cGAN trained and tested on the AMROA dataset.

Fig. 6 shows the qualitative comparison of the effect of using different patch sizes in the discriminator network, while the corresponding DSCs are listed in Table 8. The cGAN trained with the 1 × 1 PatchGAN (PixelGAN) achieved the highest segmentation accuracy for most tissues except for femoral and tibial cartilage and both muscle tissues, which were best segmented by the 34 × 34 PatchGAN. Increasing the receptive field size increases the number of parameters in the discriminator network and therefore may be more difficult to train. Additionally, as in the ‘pix2pix’ paper (Isola et al., 2017), we also noticed the repetitive tiling / checkerboard artefact (Fig. 7). However, in our instance, the artefacts become more pronounced with every increase in patch size instead of the inverse tendency as seen by (Isola et al., 2017). This could be a result of us assigning the cGANs with the reverse task (image to label) compared to the one performed by (Isola et al., 2017) (label to image).

**Table 8:** Results of PatchGAN Receptive Field Size. Comparison of segmentation performance of the proposed cGAN with different N x N receptive field sizes of the PatchGAN discriminator network. Highest DSCs achieved for each tissue are in bold.

PatchGAN Receptive Field Size Results										
Receptive Field Size	F Bone	T Bone	P Bone	F Cartilage	T Cartilage	P Cartilage	VM Muscle	GM Muscle	ACL	PCL
1 × 1	**0.971 ± 0.005**	**0.953 ± 0.012**	**0.947 ± 0.007**	0.849 ± 0.046	**0.804 ± 0.024**	**0.869 ± 0.053**	0.812 ± 0.066	0.869 ± 0.069	0.618 ± 0.140	**0.613 ± 0.143**
34 × 34	0.968 ± 0.007	0.952 ± 0.015	0.941 ± 0.013	**0.849 ± 0.002**	0.795 ± 0.013	0.868 ± 0.023	**0.883 ± 0.007**	**0.876 ± 0.009**	**0.621 ± 0.096**	0.594 ± 0.118
70 × 70	0.918 ± 0.011	0.948 ± 0.018	0.928 ± 0.002	0.812 ± 0.002	0.748 ± 0.042	0.863 ± 0.043	0.113 ± 0.085	0.000 ± 0.000	0.577 ± 0.020	0.073 ± 0.103
286 × 286	0.941 ± 0.000	0.938 ± 0.008	0.920 ± 0.012	0.766 ± 0.020	0.731 ± 0.003	0.767 ± 0.049	0.702 ± 0.022	0.597 ± 0.078	0.383 ± 0.090	0.070 ± 0.022

The cGANs were trained with the $L_{\mathrm{cGAN}}+\lambda L_{L1}$ objective with λ = 100 with training and testing being performed on the AMROA dataset.

DSCs are presented as mean ± standard deviation.

Abbreviations: FB – femoral bone, TB – tibial bone, PB – patellar bone, FC – femoral cartilage, TC – tibial cartilage, PC – patellar cartilage, VM Muscle - vastus medialis muscle, GM Muscle – medial head of gastrocnemius medialis muscle, ACL – anterior cruciate ligament, PCL – posterior cruciate ligament, DSC - Sørensen–Dice similarity coefficient.

**Fig. 8:**
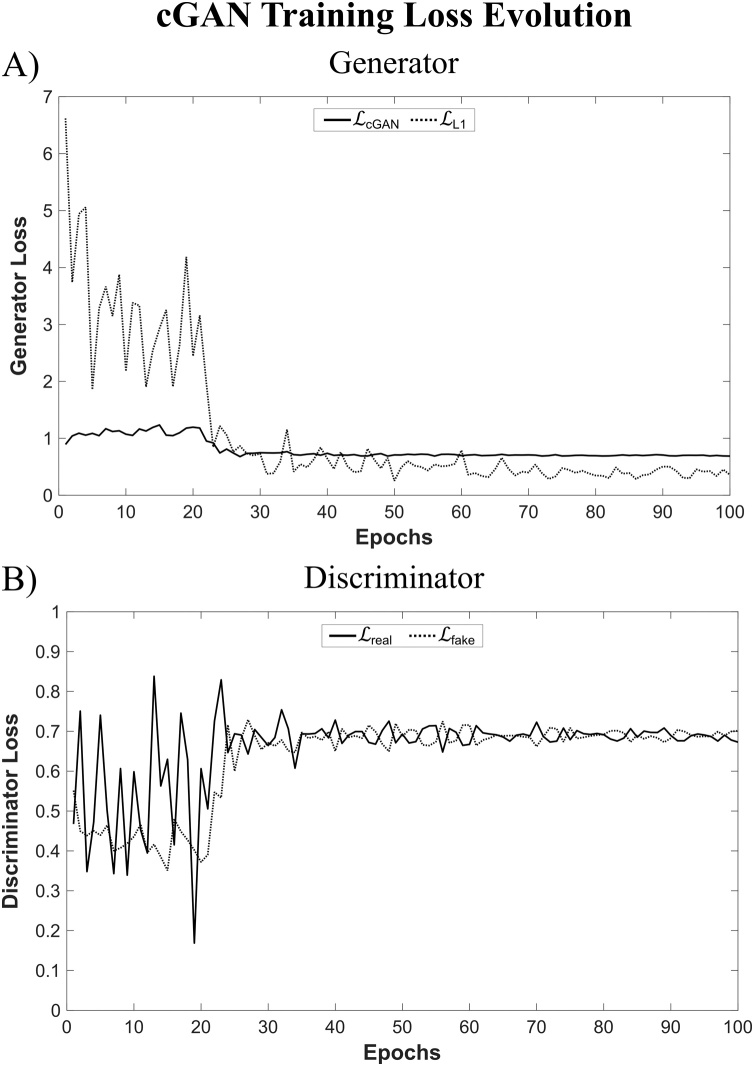
Loss Evolution during cGAN Training. The loss evolutions of the A) generator ($L_{cGAN}$ and $L_{L1}$) and B) discriminator ($L_{real}$ and $L_{fake}$) are shown for a cGAN trained with a U-Net generator and a 1 × 1 PatchGAN discriminator for 100 epochs.

Fig. 8 depicts the loss evolution during network training of the cGAN trained with the 1 × 1 PatchGAN discriminator. The loss evolutions of the cGAN generator ($L_{cGAN}$ and $L_{L1}$) and discriminator ($L_{real}$ and $L_{fake}$) are shown in Fig. 8A and B, respectively. Fig. 8B highlights how the Nash equilibrium was reached for the discriminator network during cGAN training.

### 3.6 Evaluation of transfer learning

**Fig. 9:**
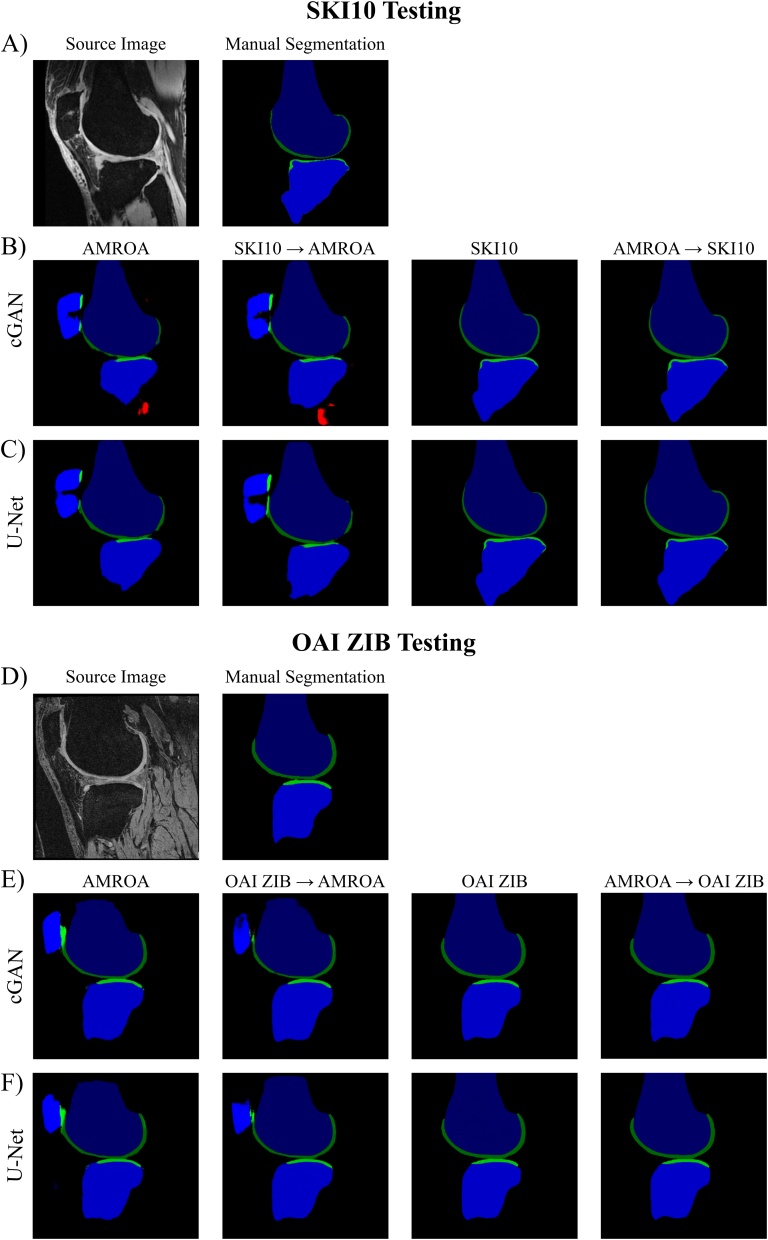
Results of Transfer Learning: SKI10 and OAI ZIB. Assessing the influence of transfer learning on segmentation performance of cGAN and U-Net when tested on the SKI10 and OAI ZIB test datasets. SKI10 / OAI ZIB → AMROA: Pretraining the network for 20 epochs on the SKI10 / OAI ZIB training dataset followed by network fine-tuning for 80 epochs on the AMROA training dataset. AMROA → SKI10 / OAI ZIB: Pretraining the network for 20 epochs on the AMROA training dataset followed by network fine-tuning for 80 epochs on the SKI10 / OAI ZIB training dataset.

**Fig. 10:**
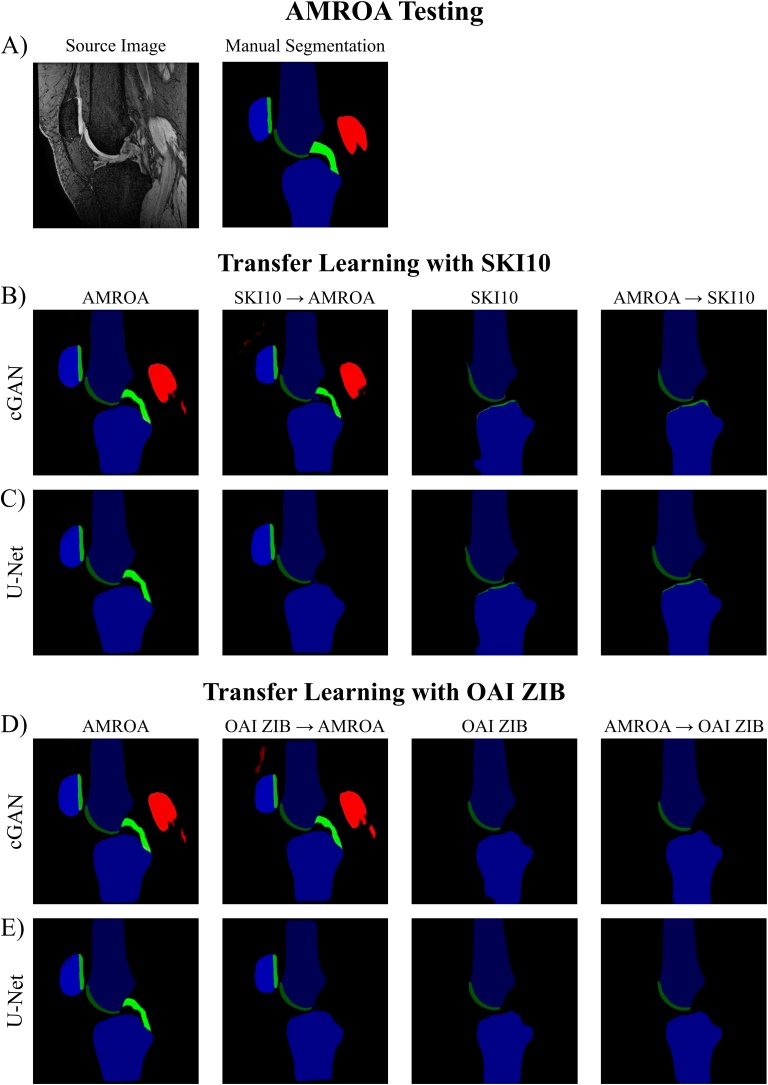
Results of Transfer Learning: AMROA. Assessing the influence of transfer learning on segmentation performance of cGAN and U-Net when tested on the AMROA test datasets. SKI10 / OAI ZIB → AMROA: Pretraining the network for 20 epochs on the SKI10 / OAI ZIB training dataset followed by network fine-tuning for 80 epochs on the AMROA training dataset. AMROA → SKI10 / OAI ZIB: Pretraining the network for 20 epochs on the AMROA training dataset followed by network fine-tuning for 80 epochs on the SKI10 / OAI ZIB training dataset.

The quantitative results of this section are presented in Table 9, Table 10 with qualitative comparisons between single step (one dataset) and two step training (transfer learning) displayed in Fig. 9, Fig. 10.

**Table 9:** Results of Transfer Learning. Comparison of segmentation performance of the proposed cGAN and U-Net without and with transfer learning and testing on the SKI10 and OAI ZIB testing dataset. Highest network scores achieved for each tissue are in bold.

Transfer Learning Results											
SKI10 Testing											
Network		Training		F Bone		T Bone		F Cartilage		T Cartilage	
				DSC	ASD	DSC	ASD	DSC	VOE	DSC	VOE
cGAN		AMROA		0.929 ± 0.040	3.726 ± 1.758	0.893 ± 0.069	3.368 ± 1.935	0.488 ± 0.093	67.19 ± 8.36	0.465 ± 0.114	69.01 ± 10.00
		SKI10		0.974 ± 0.013	1.445 ± 1.918	0.979 ± 0.007	0.527 ± 0.403	0.736 ± 0.058	41.49 ± 6.99	**0.684 ± 0.070**	**47.58 ± 7.98**
		SKI10 → AMROA		0.938 ± 0.039	3.229 ± 1.776	0.929 ± 0.041	2.696 ± 2.326	0.544 ± 0.077	62.23 ± 7.45	0.480 ± 0.100	67.86 ± 8.89
		AMROA → SKI10		0.974 ± 0.012	1.280 ± 1.484	0.977 ± 0.010	0.802 ± 1.139	**0.738 ± 0.059**	**41.19 ± 7.08**	0.675 ± 0.071	48.65 ± 7.94
U-Net		AMROA		0.925 ± 0.038	1.856 ± 0.997	0.907 ± 0.055	1.868 ± 1.336	0.545 ± 0.082	62.16 ± 7.62	0.462 ± 0.112	69.26 ± 9.86
		SKI10		0.973 ± 0.015	0.756 ± 0.995	0.978 ± 0.008	**0.254 ± 0.340**	0.728 ± 0.058	42.42 ± 6.88	0.674 ± 0.066	48.85 ± 7.55
		SKI10 → AMROA		0.943 ± 0.032	1.071 ± 0.682	0.936 ± 0.038	1.436 ± 1.083	0.576 ± 0.078	59.18 ± 7.86	0.456 ± 0.115	69.76 ± 9.93
		AMROA → SKI10		**0.975 ± 0.013**	**0.440 ± 0.492**	**0.979 ± 0.007**	0.258 ± 0.288	0.731 ± 0.056	42.08 ± 6.74	0.670 ± 0.070	49.19 ± 7.84
OAI ZIB Testing											
cGAN		AMROA		0.939 ± 0.016	4.153 ± 1.962	0.914 ± 0.080	4.681 ± 3.197	0.611 ± 0.068	55.66 ± 7.10	0.601 ± 0.089	56.44 ± 9.14
		OAI ZIB		**0.985 ± 0.002**	**0.328 ± 0.123**	0.985 ± 0.003	0.293 ± 0.072	0.895 ± 0.023	18.92 ± 3.64	**0.839 ± 0.040**	**27.55 ± 5.90**
		OAI ZIB → AMROA		0.961 ± 0.009	1.786 ± 1.202	0.961 ± 0.018	4.426 ± 2.902	0.641 ± 0.071	52.41 ± 7.87	0.738 ± 0.055	41.23 ± 6.70
		AMROA → OAI ZIB		0.985 ± 0.002	0.403 ± 0.268	**0.985 ± 0.003**	**0.293 ± 0.068**	**0.897 ± 0.022**	**18.68 ± 3.57**	0.837 ± 0.042	27.82 ± 6.19
U-Net		AMROA		0.934 ± 0.015	5.424 ± 2.799	0.915 ± 0.094	6.282 ± 3.647	0.643 ± 0.065	52.26 ± 7.03	0.626 ± 0.063	54.12 ± 6.74
		OAI ZIB		0.985 ± 0.002	0.388 ± 0.169	0.984 ± 0.003	0.304 ± 0.079	0.896 ± 0.020	18.83 ± 3.19	0.837 ± 0.038	27.80 ± 5.57
		OAI ZIB → AMROA		0.966 ± 0.006	1.244 ± 0.791	0.961 ± 0.017	1.880 ± 1.133	0.734 ± 0.046	41.83 ± 5.82	0.741 ± 0.058	40.83 ± 6.97
		AMROA → OAI ZIB		0.985 ± 0.002	0.390 ± 0.361	0.985 ± 0.003	0.327 ± 0.127	0.893 ± 0.023	19.24 ± 3.64	0.838 ± 0.037	27.75 ± 5.50

SKI10/OAI ZIB → AMROA: Pretraining the network for 20 epochs on the SKI10/OAI ZIB dataset followed by network fine-tuning for 80 epochs on the AMROA dataset.

AMROA → SKI10/OAI ZIB: Pretraining the network for 20 epochs on the AMROA dataset followed by network fine-tuning for 80 epochs on the SKI10/OAI ZIB dataset.

Results are presented as mean ± standard deviation.

Abbreviations: FB – femoral bone, TB – tibial bone, FC – femoral cartilage, TC – tibial cartilage, DSC - Sørensen–Dice similarity coefficient, ASD – average surface distance, VOE – volumetric overlap error.

**Table 10:** Results of Transfer Learning. Comparison of segmentation performance of the proposed cGAN and U-Net without and with transfer learning and testing on the AMROA testing dataset. Highest DSCs achieved for each tissue are in bold.

Transfer Learning Results											
AMROA Testing											
Network	Training	F Bone	T Bone	P Bone	F Cartilage	T Cartilage	P Cartilage	VM Muscle	GM Muscle	ACL	PCL
cGAN	AMROA	0.971 ± 0.005	0.953 ± 0.012	0.947 ± 0.007	0.849 ± 0.046	0.804 ± 0.024	0.869 ± 0.053	0.812 ± 0.066	0.869 ± 0.069	0.618 ± 0.140	0.613 ± 0.143
	SKI10	0.940 ± 0.024	0.947 ± 0.013		0.735 ± 0.005	0.561 ± 0.190					
	OAI ZIB	0.962 ± 0.009	0.951 ± 0.010		0.817 ± 0.032	0.790 ± 0.014					
	SKI10 → AMROA	0.970 ± 0.008	0.961 ± 0.004	0.940 ± 0.001	0.871 ± 0.029	0.774 ± 0.039	0.858 ± 0.038	**0.922 ± 0.037**	0.897 ± 0.057	0.586 ± 0.043	0.468 ± 0.186
	OAI ZIB → AMROA	0.972 ± 0.003	0.962 ± 0.001	0.947 ± 0.001	0.875 ± 0.026	0.811 ± 0.042	0.879 ± 0.022	0.908 ± 0.053	**0.909 ± 0.077**	0.664 ± 0.058	**0.652 ± 0.112**
	AMROA → SKI10	0.954 ± 0.015	0.949 ± 0.005		0.761 ± 0.025	0.544 ± 0.085					
	AMROA → OAI ZIB	0.960 ± 0.007	0.951 ± 0.012		0.821 ± 0.042	0.815 ± 0.015					

U-Net	AMROA	0.972 ± 0.006	0.960 ± 0.001	0.941 ± 0.010	0.886 ± 0.007	**0.834 ± 0.010**	0.890 ± 0.034	0.000 ± 0.000	0.000 ± 0.000	0.643 ± 0.153	0.641 ± 0.008
	SKI10	0.937 ± 0.031	0.944 ± 0.026		0.754 ± 0.009	0.637 ± 0.044					
	OAI ZIB	0.959 ± 0.003	0.953 ± 0.010		0.820 ± 0.026	0.798 ± 0.012					
	SKI10 → AMROA	**0.974 ± 0.003**	**0.965 ± 0.000**	0.947 ± 0.004	0.879 ± 0.012	0.815 ± 0.016	0.896 ± 0.031	0.000 ± 0.000	0.000 ± 0.000	**0.665 ± 0.114**	0.000 ± 0.000
	OAI ZIB → AMROA	0.973 ± 0.004	0.964 ± 0.005	**0.948 ± 0.005**	**0.893 ± 0.010**	0.817 ± 0.043	**0.898 ± 0.011**	0.000 ± 0.000	0.000 ± 0.000	0.648 ± 0.104	0.000 ± 0.000
	AMROA → SKI10	0.950 ± 0.031	0.959 ± 0.002		0.758 ± 0.010	0.681 ± 0.009					
	AMROA → OAI ZIB	0.962 ± 0.006	0.951 ± 0.010		0.813 ± 0.032	0.790 ± 0.039					

SKI10/OAI ZIB → AMROA: Pretraining the network for 20 epochs on the SKI10/OAI ZIB dataset followed by network fine-tuning for 80 epochs on the AMROA dataset.

AMROA → SKI10/OAI ZIB: Pretraining the network for 20 epochs on the AMROA dataset followed by network fine-tuning for 80 epochs on the SKI10/OAI ZIB dataset.

Abbreviations: FB – femoral bone, TB – tibial bone, PB – patellar bone, FC – femoral cartilage, TC – tibial cartilage, PC – patellar cartilage, VM Muscle - vastus medialis muscle, GM Muscle – medial head of gastrocnemius medialis muscle, ACL – anterior cruciate ligament, PCL – posterior cruciate ligament, DSC - Sørensen–Dice similarity coefficient.

When comparing the segmentation performances of the proposed cGAN and U-Net without and with transfer learning and testing on the SKI10 testing dataset (Table 9, Fig. 9A-C), the AMROA-pretrained / SKI10-retrained (AMROA → SKI10) U-Net showed the highest DSC scores for femoral and tibial bone and the highest boundary accuracy (i.e. smallest ASDs) for femoral bone, while the SKI10-only trained U-Net segmented the tibial bone with the highest boundary accuracy. Femoral cartilage was best segmented by the AMROA-pretrained / SKI10-retrained (AMROA → SKI10) cGAN and tibial cartilage by the SKI10-only trained cGAN.

Testing the OAI ZIB testing dataset on the proposed cGAN and U-Net without and with transfer learning (Table 9, Fig. 9D-F), the AMROA-pretrained / OAI ZIB-retrained (AMROA → OAI ZIB) cGAN showed the highest accuracies for tibial bone and femoral cartilage, while the OAI ZIB-only trained cGAN segmented the femoral bone and tibial cartilage with the highest accuracies.

When testing the cGANs and U-Nets on the AMROA testing dataset (Table 10, Fig. 10), the SKI10-pretrained / AMROA-retrained (SKI10 → AMROA) U-Net had the highest DSCs for femoral and tibial bone as well as the ACL. Femoral cartilage as well as patellar bone and cartilage was segmented most accurately by the OAI ZIB-pretrained / AMROA-retrained (OAI ZIB → AMROA) U-Net. The AMROA only trained U-Net showed the best segmentation accuracy for tibial cartilages. The SKI10-pretrained / AMROA-retrained (SKI10 → AMROA) cGAN provided the highest segmentation score for the vastus medialis muscle while the medial head of gastrocnemius muscle and the PCL was best segmented by the OAI ZIB-pretrained / AMROA-retrained (OAI ZIB → AMROA) cGAN. Compared to the U-Net, the cGAN could successfully segment both medial muscles which could promote a strength of the cGAN. A further note is that, although the SKI10 and OAI ZIB datasets only comprised of segmentations of femoral and tibial bone and cartilage, the cGANs and U-Nets initialised with the respective SKI10- and OAI ZIB-pretrained network weights and retrained on the AMROA dataset were able to recuperate and capture patellar, ligament and muscle tissues.

A challenge of any machine learning technique is obtaining a training set that optimises the amount of variation from the rare morphology of pathological conditions or image artefacts. The AMROA dataset was highly controlled, with the patients and imaging occurring with a single imaging protocol on a single MRI system. The images showed a clear bone-cartilage separation and enabled better cartilage segmentation scores after training than the SKI10 dataset. The OAI ZIB dataset highlights the benefits of training on a very large number of images with the cGAN and U-Net (OAI ZIB-only trained) achieving DSC ≥ 0.984 for bone and DSC ≥ 0.837 for cartilage segmentations.

The ability for the network to be used under variable conditions was simulated by using three knee datasets (AMROA, SKI10 and OAI ZIB). Even without transfer learning, the AMROA training enabled SKI10 and OAI ZIB segmentation and vice versa, albeit not with high accuracy, but nonetheless indicating the robustness of deep learning methods. Transfer learning not only improved the segmentation accuracy for some tissues of the local dataset but also enhanced the networks ability to segment the SKI10 / OIA ZIB test dataset by introducing more heterogeneity into the model. Even though the SKI10- and OAI ZIB-pretrained networks were then fine-tuned to segment the local AMROA dataset, it could segment the SKI10 and OAI ZIB testing dataset with an improved performance compared to the AMROA-only trained network without pretraining. This effect was seen for both cGANs and U-Nets.

### 3.7 AMROA: comparison to previous studies

In this subsection, the results obtained for the different tissues semantically segmented in this study are compared to those of previous studies. The cGAN and U-Net achieving the highest segmentation accuracy on the AMROA dataset for each respective tissue is chosen for this purpose.

#### 3.7.1 Bone

While cartilage has been traditionally studied for OA, bone shape has been under increasing investigations (Ambellan et al., 2019; Felson and Neogi, 2004). Bone shape has been linked to radiographic OA (Hunter et al., 2015; Neogi et al., 2013; Wise et al., 2018) and associated with longitudinal pain progression (Hunter et al., 2015). Segmented bone can be used to separate out bone-specific diseases, such as osteochondral defects.

The OAI ZIB-pretrained / AMROA-retrained cGAN trained with the $L_{cGAN}+\lambda L_{L1}$ loss objective (λ = 100) and a 1 × 1 PixelGAN generated segmentations of femoral (DSC = 0.972), tibial (DSC = 0.962) and patellar (DSC = 0.947) bone with the highest accuracy. The SKI10-pretrained / AMROA-retrained U-Net ($L_{L1}$ loss objective) achieved slightly higher segmentation accuracies for femoral and tibial bone tissues (femoral: DSC = 0.974; tibial: DSC = 0.965) and the OAI ZIB-pretrained / AMROA-retrained U-Net for patellar bone (DSC = 0.948), compared to the cGANs. The boundaries of the images, near the top and bottom of any 2D slice, did not always segment all bone, which is where the MRI radiofrequency (RF) transmit and receive uniformity was poor due to characteristics of the MRI coil. Traditional semi-automatic approaches involving signal threshold, region-based or clustering segmentation can be similarly sensitive to image non-uniformities (Swanson et al., 2010). These non-uniformities are shown as a change in signal-to-noise or darkening of the surrounding muscle tissues (see lower regions of Fig. 2). These effects from RF transmit or receive non-uniformity could be mitigated with a larger training population, as more complex modelling of data is possible. Nevertheless, segmentation of the patella achieved the lowest accuracy. The patella has the widest range of inter-subject variability when compared to the larger tibial and femoral bones. The patella bone can vary in both shape and position, shifting due to the orientation and bend of the knee. Additionally, due to its smaller volume, fewer training images are used for the patella segmentation.

The cGAN and U-Net bone segmentation scores achieved in this study are similar to those achieved by a CycleGAN method using unannotated knee MR images for femoral (DSC = 0.95 – 0.97) and tibial (DSC = 0.93 – 0.95) bone segmentation (Liu, 2019), and a convolutional encoder-decoder network combined with a 3D fully connected conditional random field and simplex deformable modelling for femoral (DSC = 0.970), tibial (DSC = 0.962) and patellar (DSC = 0.898) bone segmentation (Zhou et al., 2018).

#### 3.7.2 Cartilage

For a long time, OA was considered a disease primarily involving variations in articular cartilage composition and morphology. Therefore, the attention was predominantly placed on the extraction of OA biomarkers from quantitative MR imaging techniques using manual or semi-manual segmentation techniques that suffer from intra- and inter-observer variability (Pedoia et al., 2016). Deep learning methods can provide a fast and repeatable alternative to overcome these time-consuming and operator-dependent procedures.

The OAI ZIB-pretrained / AMROA-retrained cGAN trained with the $L_{cGAN}+\lambda L_{L1}$ loss objective (λ = 100) and a 1 × 1 PixelGAN generated segmentations of femoral (DSC = 0.875), tibial (DSC = 0.811) and patellar (DSC = 0.879) cartilage with the highest accuracy from all cGAN trainings. The OAI ZIB-pretrained / AMROA-retrained U-Net ($L_{L1}$ loss objective) achieved marginally higher accuracies for femoral (DSC = 0.893) and patellar (DSC = 0.898) cartilage segmentations and the AMROA-only trained U-Net ($L_{L1}$ loss objective) achieved a slightly higher segmentation accuracy for tibial cartilage (DSC = 0.834) compared to the cGAN results.

The cartilage segmentation performances of both cGAN and U-Net are comparable to those attained by a 2D U-Net for femoral, tibial and patellar cartilage segmentations on T1ρ-weighted (DSC = 0.632−0.702) and DESS MR images (DSC = 0.767−0.878) (Norman et al., 2018), a CycleGAN method for femoral and tibial cartilage segmentation on PD-weighted (DSC = 0.65−0.66) and T2-weighted FSE images (DSC = 0.81−0.75) (Liu, 2019), as well as the recently investigated cGAN for femoral, tibial and patellar segmentation on DESS MR images (DSC = 0.843−0.918) (Gaj et al., 2019).

#### 3.7.3 Muscle

As muscle weakness and atrophy can be regarded as preceding risk factors and resulting pain-related consequences for the development and progression of OA, studying morphological changes in knee joint muscles has become increasingly important (Fink et al., 2007; Slemenda et al., 1997).

The SKI10-pretrained / AMROA-retrained cGAN and the OAI ZIB-pretrained / AMROA-retrained cGAN trained with the $L_{cGAN}+\lambda L_{L1}$ loss objective (λ = 100) and a 1 × 1 PixelGAN segmented the medial gastrocnemius muscle (DSC = 0.909) and medial vastus muscle (DSC = 0.922) with the highest accuracies, respectively. The U-Net trained with altered loss objective ($L_{L2}$ → $L_{L1}$) achieved the highest segmentation accuracies for both the medial gastrocnemius (DSC = 0.933) and vastus (DSC = 0.914) muscles.

Our results are comparatively lower compared to those of a semi-automatic single-atlas (DSC = 0.95−0.96) and fully-automatic multi-atlas (DSC = 0.91 – 0.94) based approach for medial vastus segmentation (Le Troter et al., 2016), and a 2D U-Net for quadriceps (DSC = 0.98) segmentation (Kemnitz et al., 2019). A crucial difference between these studies and ours is the plane in which segmentation was performed. While muscles are typically segmented on axial images as this provides a more straightforward task with clearer separation between different muscles, our multi-class tissue segmentation approach was performed on sagittal images. Segmenting different muscles in the sagittal plane is a demanding task, especially in areas of the calf muscles where the two-headed gastrocnemius muscle overlaps (medial and lateral) while also overlaying the soleus muscle.

#### 3.7.4 Cruciate ligament

There has been a growing interest in investigating and understanding the mechanism responsible for the post-traumatic development of OA following injury to the cruciate ligaments, especially the ACL (Chaudhari et al., 2008; Messer et al., 2019; Monu et al., 2017). Although ACL reconstruction and rehabilitation can help restore patients to normal life and previous activities, it cannot prevent the long-term risk of developing OA (Paschos, 2017). Accurate and repeatable segmentations of the cruciate ligaments are required when aiming at evaluating longitudinal changes in the cruciate ligaments following reconstructive surgery.

In our study, the OAI ZIB-pretrained / AMROA-retrained cGAN trained with the 1 × 1 PixelGAN and $L_{cGAN}+\lambda L_{L1}$ loss objective (λ = 100) achieved the highest accuracy for ACL (DSC = 0.664) and PCL segmentation (DSC = 0.652). The SKI10-pretrained / AMROA-retrained U-Net ($L_{L1}$ loss objective) achieved a similar accuracy for ACL segmentation (DSC = 0.665) and the AMROA-only trained U-Net ($L_{L1}$ loss objective) achieved a marginally lower accuracy for PCL segmentation (DSC = 0.641), compared to the best performing cGANs.

(Lee et al., 2013) proposed a graph cut method for automatic ACL segmentation and attained a DSC score of 0.672, while (Paproki et al., 2016) used a patch-based method for PCL segmentation to achieve a DSC score of 0.744. Using a 3D convolutional neural network (CNN), (Mallya et al., 2019) achieved DSC scores of 0.40 and 0.61 for ACL and PCL segmentations, respectively. When combining their 3D CNN with a deformable atlas-based segmentation method, their ACL (DSC = 0.84) and PCL (0.85) segmentation accuracies increased substantially. In general, 3D networks could provide higher segmentation accuracies especially for fine structures such as the cruciate ligaments that only appear on a few 2D slices in a 3D dataset. However, 2D segmentation techniques are useful for broader applicability, as 2D imaging is often faster and currently still more clinically employed than 3D imaging.

The lower similarity scores achieved in our study compared to the other studies could arise from the use of 3D-FS SPGR images as source images during training as these are non-optimal for the segmentation of the cruciate ligaments due to their less than ideal soft tissue separation with surrounding structures and fluid. Fat-saturated proton-density-weighted fast spin echo or T2-weighted fast spin echo images are more suitable for segmentation purposes as shown by (Mallya et al., 2019) and (Paproki et al., 2016), respectively. These sequences are clinically used for cruciate ligament assessment due to their dark appearance and clear separation from fluid and other surrounding tissues.

### 3.8 SKI10 and OAI ZIB: comparison to previous studies

In this subsection, the segmentation results of the SKI10 and OAI ZIB datasets in this study are compared to those of previous studies. The cGAN and U-Net achieving the highest segmentation accuracy on these datasets is chosen for this purpose.

#### 3.8.1 SKI10

The AMROA-pretrained / SKI10-retrained U-Net ($L_{L1}$ loss objective) achieved a comparable ASD score for femoral bone (ASD = 0.44 mm) and an improved ASD score for tibial bone (ASD = 0.26 mm) to those reported by (Liu et al., 2017) and (Ambellan et al., 2019). However, the segmentation accuracies for femoral (VOE ≥ 42.2 %) and tibial (VOE ≥ 47.6 %) cartilage achieved by our models were substantially lower.

#### 3.8.2 OAI ZIB

The OAI ZIB-only trained cGAN trained with the $L_{cGAN}+\lambda L_{L1}$ loss objective (λ = 100) and a 1 × 1 PixelGAN generated segmentations of femoral bone (DSC = 0.985) and tibial cartilage (DSC = 0.839) with the highest accuracy. AMROA-pretrained / OAI ZIB-retrained cGAN trained with the 1 × 1 PixelGAN and $L_{cGAN}+\lambda L_{L1}$ loss objective (λ = 100) achieved the highest accuracy for tibial bone (DSC = 0.985) and femoral cartilage (DSC = 0.897) segmentation. The ASD of both the femoral (ASD = 0.33 mm) and tibial (ASD = 0.29 mm) bones were smaller than image resolution of the OAI DESS images (0.36 × 0.36 × 0.7 mm^3^). Although we achieve similar DSC scores for all tissues on the OAI ZIB dataset compared to those presented in (Ambellan et al., 2019), our ASD scores were larger. The pixel-wise error losses ($L_{L1}$. $L_{L2}$ and $L_{SmL1}$) used to train the networks in our work were chosen to maintain an effective comparison between the cGAN and the U-Net. However, training our models with loss functions more traditionally used for segmentation purposes such as multi-class Dice similarity or cross entropy might lead to more comparable results for boundary-distance-based metrics.

### 3.9 Limitations

The network performances are depended on the accuracy of the ground truth segmentations. Inaccuracies or errors in the segmentation maps could result in a less accurate network, especially when trained on a low number of image volumes, as done in this study. Additionally, training a network on a low number of high-quality images restricts the networks applicability to only highly controlled studies with homogeneous data. Therefore, the networks trained in this study might be limited in their application in clinical settings where high image quality is not always achievable due to patient conditions and operator variabilities.

Network training on 2D MR image slices is considerably less computationally demanding than on 3D volumes. For the purposes of this study such as investigating the effects of training with different loss objectives and cGAN discriminator networks, it was sufficient to train on 2D images. Nevertheless, the segmentation of small knee joint structures, such as the cruciate ligaments, could benefit from 3D networks that should add spatial continuity along the slice dimension.

Furthermore, the segmentation results presented in this study are from standalone networks without further processing within a pipeline. Therefore, the obtained results, especially for cartilage segmentation, are not comparable to those from current state-of-the-art pipeline methods such as described by (Liu et al., 2017) and (Ambellan et al., 2019) that initially perform automated segmentation using a CNN followed by further refinement using deformable or statistical shape models, respectively.

Lastly, additional investigations into varying the network architectures and optimisation strategies are warranted, with ever more loss functions as well as layer combination and optimisation strategies continuously being developed.

## 4 Conclusion

This work demonstrated the usage of a cGAN, using a U-Net generator with a PatchGAN discriminator, for the purpose of automatically segmenting multiple knee joint tissues on MR images. While DSC > 0.95 were achieved for all segmented bone structures and DSC > 0.83 for cartilage and muscle tissues, DSC of only ≈0.66 were achieved for cruciate ligament segmentations. Nevertheless, this segmentation performance was attained despite the low number of subjects (N = 8) for training on the local dataset. Although the U-Net outperformed the cGAN in most knee joint tissue segmentations, this study provides an optimal platform for future technical developments for utilising cGANs for segmentation tasks. By enabling automated and simultaneous segmentation of multiple tissues we hope to increase the accuracy and time efficiency for evaluating joint health in osteoarthritis.

## CRediT authorship contribution statement

**Dimitri A. Kessler:** Conceptualization, Methodology, Software, Investigation, Data curation, Visualization, Writing - original draft. **James W. MacKay:** Conceptualization, Data curation, Writing - review & editing. **Victoria A. Crowe:** Data curation, Writing - review & editing. **Frances M.D. Henson:** Resources, Writing - review & editing. **Martin J. Graves:** Resources, Writing - review & editing. **Fiona J. Gilbert:** Conceptualization, Writing - review & editing, Supervision. **Joshua D. Kaggie:** Conceptualization, Methodology, Software, Writing - original draft, Supervision.

## Declaration of Competing Interest

The authors report no declarations of interest.

## Figures and Tables

**Table:** 

Training time (s/epoch): AMROA:	135 (cGAN with 1 × 1 PixelGAN)
	130 (cGAN with 70 × 70 PatchGAN)
	100 (U-Net)
SKI10:	380 (cGAN with 1 × 1 PixelGAN)
	210 (U-Net)
OAI ZIB:	2710 (cGAN with 1 × 1 PixelGAN)
	1530 (U-Net)

**Table:** 

Training time (s/epoch): AMROA:	110 (cGAN with 70 × 70 PatchGAN)
90 (U-Net)	

**Table:** 

Training time (s/epoch): AMROA:	120 (cGAN with 70 × 70 PatchGAN)
100 (U-Net)	

**Table:** 

Training time (s/epoch): AMROA:	105 (cGAN with 70 × 70 PatchGAN)
70 (U-Net)	

**Table:** 

Training time (s/epoch): AMROA:	100 (cGAN with 70 × 70 PatchGAN)
75 (U-Net)	

**Table:** 

Training time (s/epoch): AMROA:	245 (cGAN with 70 × 70 PatchGAN)
220 (U-Net)	

## Appendix A

### Network Description

Generator: The encoding part of the generator network consists of the repeated application of nine 4 × 4 convolutions with stride 2, down-sampling the input by a factor of 2 at each layer. Each convolution is followed by a batch normalisation layer (except the first layer) and a leaky rectified linear unit (leaky ReLU) with slope 0.2. During the first encoding step the number of feature channels is changed from 3 to 64. At the subsequent three encoding steps, the number of feature channels is doubled (64–512), while the following five are kept at 512. In the ensuing decoding part, the input is repeatedly up-sampled by a factor of 2 by nine 4 × 4 transpose convolutional layers with stride 2 and additional skip connections (concatenations) between each layer i and 9-i, changing the number of feature channels at each step. The first four decoder convolutions are followed by batch normalisation, dropout (50 %) and a ReLU. The next four decoder convolutions are followed by batch normalisation and a ReLU without dropout. After the final layer a convolution followed by a Tanh activation layer is applied to generate the segmentation map.

Total number of parameters: 66.999 M

Generator with five convolutions in encoder/decoder: In this generator network, the encoding part consists of the repeated application of five 4 × 4 convolutions with stride 2, down-sampling the input by a factor of 2 at each layer. In the ensuing decoding part, the input is repeatedly up-sampled by a factor of 2 by five 4 × 4 transpose convolutional layers with stride 2 and additional skip connections between each layer i and 5-i.

Total number of parameters: 16.659 M

Generator with seven convolutions in encoder/decoder: The encoding part consists of the repeated application of seven 4 × 4 convolutions with stride 2, down-sampling the input by a factor of 2 at each layer. In the subsequent decoding part, the input is repeatedly up-sampled by a factor of 2 by seven 4 × 4 transpose convolutional layers with stride 2 and additional skip connections between each layer i and 7-i.

Total number of parameters: 41.829 M

Generator with 16 as minimum number of feature channels: In this network, the number of feature channels is changed from 3 to 16 during the first encoding step. During the following three encoding steps, the number of feature channels is doubled (16–128), while the subsequent five are kept at 128.

Total number of parameters: 4.191 M

Generator with 32 as minimum number of feature channels: The number of feature channels is changed from 3 to 32 during the first encoding step. In the following three encoding steps, the number of feature channels is doubled (32–256), while the subsequent five are kept at 256.

Total number of parameters: 16.755 M

Generator with 128 as minimum number of feature channels: In the first encoding step the number of feature channels is changed from 3 to 128. In the following three encoding steps, the number of feature channels is doubled (128–1024), while the subsequent five are kept at 1024.

Total number of parameters: 267.953 M

Discriminator:

70 × 70 PatchGAN: The discriminator network repeatedly down-samples the input by applying three 4 × 4 convolutions with stride 2 followed by two 4 × 4 convolutions with stride 1. Each convolution during down-sampling is followed by a batch normalisation layer (except the first and last layer) and a leaky ReLU (slope 0.2) (except for the last layer). The number of feature channels are doubled (64–512) during the first four convolutional steps. The final convolutional layer is proceeded by a Sigmoid activation layer.

Total number of parameters: 2.769 M

1 × 1 PatchGAN (PixelGAN): This PixelGAN discriminator network applies three 1 × 1 convolutions with stride 1, where the first convolution is followed by a leaky ReLU (slope 0.2), the second convolution by a batch normalisation layer and a leaky ReLU (slope 0.2) and the final convolution by a Sigmoid activation function. The number of feature channels are doubled (64–128) during the first two convolutions.

Total number of parameters: 0.009 M

34 × 34 PatchGAN: This network repetitively down-samples the input by using two 4 × 4 convolutions with stride 2 followed by two 4 × 4 convolutions with stride 1. Each convolution is followed by a batch normalisation layer (except the first and last layer) and a leaky ReLU (slope 0.2) (except for the last layer). The number of feature channels are doubled (64–256) during the first three convolutional steps. The final layer is ensued by a Sigmoid activation layer.

Total number of parameters: 0.666 M

286 × 286 PatchGAN: This discriminator network consists of eight convolutional layers with 4 × 4 spatial filters. The first 6 convolutions have stride 2 while the last two have stride 1. Each convolutional layer is followed by a batch normalisation layer (except the first and last layer) and a leaky ReLU (slope 0.2) (except for the last layer). The number of feature channels are doubled (64–512) during the first four convolutions and kept at 512 for the ensuing layers. A Sigmoid activation layer succeeds the final convolution.

Total number of parameters: 11.159 M

## References

[bib0005] Ambellan F., Tack A., Ehlke M., Zachow S. (2019). Automated segmentation of knee bone and cartilage combining statistical shape knowledge and convolutional neural networks: data from the Osteoarthritis Initiative. Med. Image Anal..

[bib0010] Benhamou C.L., Poupon S., Lespessailles E., Loiseau S., Jennane R., Siroux V., Ohley W., Pothuaud L. (2001). Fractal analysis of radiographic trabecular bone texture and bone mineral density: Two complementary parameters related to osteoporotic fractures. J. Bone Miner. Res..

[bib0015] Bindernagel M., Kainmueller D., Seim H., Lamecker H., Zachow S., Hege H.C. (2011). An articulated statistical shape model of the human knee. Inform. aktuell.

[bib0020] Blumenkrantz G., Majumdar S. (2016). Quantitative magnetic resonance imaging of articular. Eur. Cells Mater..

[bib0025] Chaudhari A.M.W., Briant P.L., Bevill S.L., Koo S., Andriacchi T.P. (2008). Knee kinematics, cartilage morphology, and osteoarthritis after ACL injury. Med. Sci. Sports Exerc..

[bib0030] Chen C., Dou Q., Chen H., Heng P.A. (2018). Semantic-aware generative adversarial nets for unsupervised domain adaptation in chest X-ray segmentation. Lect. Notes Comput. Sci. (including Subser. Lect. Notes Artif. Intell. Lect. Notes Bioinformatics).

[bib0035] Deniz C.M., Xiang S., Hallyburton R.S., Welbeck A., Babb J.S., Honig S., Cho K., Chang G. (2018). Segmentation of the proximal femur from MR images using deep convolutional neural networks. Sci. Rep..

[bib0040] Dice L.R. (1945). Measures of the amount of ecologic association between species. Ecology.

[bib0045] Dou Q., Ouyang C., Chen C., Chen H., Heng P.A. (2018). Unsupervised cross-modality domain adaptation of convnets for biomedical image segmentations with adversarial loss. IJCAI Int. Jt. Conf. Artif. Intell..

[bib0050] Felson D.T., Neogi T. (2004). Osteoarthritis: Is It a Disease of Cartilage or of Bone?. Arthritis Rheum..

[bib0055] Fink B., Egl M., Singer J., Fuerst M., Bubenheim M., Neuen-Jacob E. (2007). Morphologic changes in the vastus medialis muscle in patients with osteoarthritis of the knee. Arthritis Rheum..

[bib0060] Gaj S., Yang M., Nakamura K., Li X. (2019). Automated cartilage and meniscus segmentation of knee MRI with conditional generative adversarial networks. Magn. Reson. Med..

[bib0065] Girshick R. (2015). Fast R-CNN. Proc. IEEE Int. Conf. Comput. Vis. 2015 Inter.

[bib0070] Goldring M.B., Culley K.L., Otero M., Grässel S., Aszódi A. (2017). Pathogenesis of osteoarthritis in General. Cartilage: Volume 2: Pathophysiology.

[bib0075] Goodfellow I.J., Pouget-Abadie J., Mirza M., Xu B., Warde-Farley D., Ozair S., Courville A., Bengio Y. (2014). Generative Adversarial Networks.

[bib0080] Heimann T., Styner M., Warfield S.K. (2010). Segmentation of Knee Images : A Grand Challenge Segmentation of Knee Images. http://www.ski10.org/ski10.pdf.

[bib0085] Hunter D.J., Eckstein F. (2009). Exercise and osteoarthritis. J. Anat..

[bib0090] Hunter D., Nevitt M., Lynch J., Kraus V.B., Katz J.N., Collins J.E., Bowes M., Guermazi A., Roemer F.W., Losina E. (2015). Longitudinal validation of periarticular bone area and 3D shape as biomarkers for knee OA progression? Data from the FNIH OA Biomarkers Consortium. Ann. Rheum. Dis. annrheumdis-2015-207602..

[bib0095] Huo Y., Xu Z., Bao S., Assad A., Abramson R.G., Landman B.A. (2018). Adversarial synthesis learning enables segmentation without target modality ground truth. Proc. - Int. Symp. Biomed. Imaging.

[bib0100] Ismail H.M., Vincent T.L., Grässel S., Aszódi A. (2017). Cartilage injury and osteoarthritis. Cartilage: Volume 2: Pathophysiology.

[bib0105] Isola P., Zhu J.Y., Zhou T., Efros A.A. (2017). Image-to-image translation with conditional adversarial networks. CVPR Proc. - 30th IEEE Conf. Comput. Vis. Pattern Recognition.

[bib0110] Kemnitz J., Baumgartner C.F., Eckstein F., Chaudhari A., Ruhdorfer A., Wirth W., Eder S.K., Konukoglu E. (2019). Clinical evaluation of fully automated thigh muscle and adipose tissue segmentation using a U-Net deep learning architecture in context of osteoarthritic knee pain. Magn. Reson. Mater. Physics, Biol. Med..

[bib0115] Larobina M., Murino L. (2014). Medical image file formats. J. Digit. Imaging.

[bib0120] Le Troter A., Fouré A., Guye M., Confort-Gouny S., Mattei J.P., Gondin J., Salort-Campana E., Bendahan D. (2016). Volume measurements of individual muscles in human quadriceps femoris using atlas-based segmentation approaches. Magn. Reson. Mater. Physics, Biol. Med..

[bib0125] Lee H., Hong H., Kim J., Lee K.M., Matsushita Y., Rehg J.M., Hu Z. (2013). Anterior cruciate ligament segmentation from knee MR images using graph cuts with geometric and probabilistic shape constraints.

[bib0130] Lee J.G., Gumus S., Moon C.H., Kwoh C.K., Bae K.T. (2014). Fully automated segmentation of cartilage from the MR images of knee using a multi-atlas and local structural analysis method. Med. Phys..

[bib0135] Li C., Wand M. (2016). Precomputed Real-Time Texture Synthesis with Markovian Generative Adversarial Networks.

[bib0140] Liu F. (2019). SUSAN: segment unannotated image structure using adversarial network. Magn. Reson. Med..

[bib0145] Liu F., Zhou Z., Jang H., Samsonov A., Zhao G., Kijowski R. (2017). Deep convolutional neural network and 3D deformable approach for tissue segmentation in musculoskeletal magnetic resonance imaging. Magn. Reson. Med..

[bib0150] Lohmander L.S., Englund P.M., Dahl L.L., Roos E.M. (2007). The long-term consequence of anterior cruciate ligament and meniscus injuries: osteoarthritis. Am. J. Sports Med..

[bib0155] Long J., Shelhamer E., Darrell T. (2018). Fully convolutional adaptation networks for semantic segmentation. Proc. IEEE Comput. Soc. Conf. Comput. Vis. Pattern Recognit.

[bib0160] MacKay J.W., Kapoor G., Driban J.B., Lo G.H., McAlindon T.E., Toms A.P., McCaskie A.W., Gilbert F.J. (2018). Association of subchondral bone texture on magnetic resonance imaging with radiographic knee osteoarthritis progression: data from the osteoarthritis initiative bone ancillary study. Eur. Radiol..

[bib0165] MacKay J.W., Kaggie J.D., Treece G.M., McDonnell S.M., Khan W., Roberts A.R., Janiczek R.L., Graves M.J., Turmezei T.D., McCaskie A.W., Gilbert F.J. (2020). Three-dimensional surface-based analysis of cartilage MRI data in knee osteoarthritis: validation and initial clinical application. J. Magn. Reson. Imaging.

[bib0170] Mallya Y., J Vijayananda, M.S Vidya, Venugopal V.K., Mahajan V. (2019). Automatic delineation of anterior and posterior cruciate ligaments by combining deep learning and deformable atlas based segmentation. Med. Imaging 2019 Biomed. Appl. Mol. Struct. Funct. Imaging.

[bib0175] Martel-Pelletier J., Barr A.J., Cicuttini F.M., Conaghan P.G., Cooper C., Goldring M.B., Goldring S.R., Jones G., Teichtahl A.J., Pelletier J.P. (2016). Osteoarthritis. Nat. Rev. Dis. Prim..

[bib0180] Messer D.J., Shield A.J., Williams M.D., Timmins R.G., Bourne M.N. (2019). Hamstring muscle activation and morphology are significantly altered 1–6 years after anterior cruciate ligament reconstruction with semitendinosus graft. Knee Surg. Sports Traumatol. Arthrosc..

[bib0185] Monu U.D., Jordan C.D., Samuelson B.L., Hargreaves B.A., Gold G.E., McWalter E.J. (2017). Cluster analysis of quantitative MRI T 2 and T 1ρ relaxation times of cartilage identifies differences between healthy and ACL-injured individuals at 3T. Osteoarthr. Cartil..

[bib0190] Neogi T., Bowes M.A., Niu J., De Souza K.M., Vincent G.R., Goggins J., Zhang Y., Felson D.T. (2013). Magnetic resonance imaging-based three-dimensional bone shape of the knee predicts onset of knee osteoarthritis: data from the osteoarthritis initiative. Arthritis Rheum..

[bib0195] Ng H.P., Ong S.H., Foong K.W.C., Goh P.S., Nowinski W.L. (2006). Medical image segmentation using K-means clustering and improved watershed algorithm. IEEE Southwest Symp. Image Anal. Interpret.

[bib0200] Norman B., Pedoia V., Majumdar S. (2018). Use of 2D U-Net convolutional neural networks for automated cartilage and Meniscus segmentation of knee MR imaging data to determine relaxometry and morphometry. Radiology.

[bib0205] Paproki A., Wilson K.J., Surowiec R.K., Ho C.P., Pant A., Bourgeat P., Engstrom C., Crozier S., Fripp J. (2016). Automated segmentation and T2-mapping of the posterior cruciate ligament from MRI of the knee: data from the osteoarthritis initiative. Proc. - 2016 IEEE 13th Int. Symp. Biomed. Imaging.

[bib0210] Paschos N.K. (2017). Anterior cruciate ligament reconstruction and knee osteoarthritis. World J. Orthop..

[bib0215] Patel F.K., Singh M. (2018). Segmentation of cartilage from knee MRI images using the watershed algorithm. Int. J. Adv. Res. Ideas Innov. Technol..

[bib0220] Pathak D., Krahenbuhl P., Donahue J., Darrell T., Efros A.A. (2016). Context Encoders: Feature Learning By Inpainting.

[bib0225] Pedoia V., Majumdar S., Link T.M. (2016). Segmentation of joint and musculoskeletal tissue in the study of arthritis. Magn. Reson. Mater. Physics, Biol. Med..

[bib0230] Regmi K., Borji A. (2018). Cross-View Image Synthesis using Conditional GANs.

[bib0235] Rezaei M., Harmuth K., Gierke W., Kellermeier T., Fischer M., Yang H., Meinel C. (2017). A conditional adversarial network for semantic segmentation of brain tumor. BrainLes 2017 Brainlesion Glioma, Mult. Sclerosis, Stroke Trauma. Brain Inj. Springer.

[bib0240] Ronneberger O., Fischer P., Brox T. (2015). U-Net: Convolutional Networks for Biomedical Image Segmentation.

[bib0245] Seim H., Kainmueller D., Lamecker H., Bindernagel M., Malinowski J., Zachow S. (2010). Model-based auto-segmentation of knee bones and cartilage in MRI data. Med. Image Anal. Clin. A Gd. Chall..

[bib0250] Shan L., Zach C., Charles C., Niethammer M. (2014). Automatic atlas-based three-label cartilage segmentation from MR knee images. Med. Image Anal..

[bib0255] Shie C.K., Chuang C.H., Chou C.N., Wu M.H., Chang E.Y. (2015). Transfer representation learning for medical image analysis. Proc. Annu. Int. Conf. IEEE Eng. Med. Biol. Soc.

[bib0260] Shrivastava K., Gupta N., Sharma N. (2014). Medical image segmentation using modified K means clustering. Int. J. Comput. Appl..

[bib0265] Slemenda C., Brandt K.D., Heilman D.K., Mazzuca S., Braunstein E.M., Katz B.P., Wolinsky F.D. (1997). Quadriceps weakness and osteoarthritis of the knee. Ann. Intern. Med..

[bib0270] Sørensen T.J. (1948). A method of establishing groups of equal amplitude in plant sociology based on similarity of species and its application to analyses of the vegetation on Danish commons. Biol. Skr..

[bib0275] Swanson M.S., Prescott J.W., Best T.M., Powell K., Jackson R.D., Haq F., Gurcan M.N. (2010). Semi-automated segmentation to assess the lateral Meniscus in normal and osteoarthritic knees. Osteoarthr. Cartil..

[bib0280] The Osteoarthritis Initiative [WWW Document], n.d.. https://nda.nih.gov/oai/.

[bib0285] Treece G.M., Prager R.W., Gee A.H. (1999). Regularised marching tetrahedra: improved iso-surface extraction. Comuters Graph..

[bib0290] Wise B.L., Niu J., Zhang Y., Liu F., Pang J., Lynch J.A., Lane N.E. (2018). Bone shape mediates the relationship between sex and incident knee osteoarthritis. BMC Musculoskelet. Disord..

[bib0295] Xia Y., Fripp J., Chandra S.S., Schwarz R., Engstrom C., Crozier S. (2013). Automated bone segmentation from large field of view 3D MR images of the hip joint. Phys. Med. Biol..

[bib0300] Yang D., Xu D., Zhou K., Georgescu B., Chen M., Grbic S., Metaxas D., Comaniciu D. (2017). Automatic liver segmentation using an adversarial image-to-Image network. Med. Image Comput. Comput. Assist. Interv. − MICCAI 2017, Springer.

[bib0305] Yushkevich P.A., Piven J., Hazlett H.C., Smith R.G., Ho S., Gee J.C., Gerig G. (2006). User-guided 3D active contour segmentation of anatomical structures: significantly improved efficiency and reliability. Neuroimage.

[bib0310] Zhao J.J., Mathieu M., LeCun Y. (2017).

[bib0315] Zhou L., Chav R., Cresson T., Chartrand G., De Guise J. (2016). 3D knee segmentation based on three MRI sequences from different planes. Proc. Annu. Int. Conf. IEEE Eng. Med. Biol. Soc.

[bib0320] Zhou Z., Zhao G., Kijowski R., Liu F. (2018). Deep convolutional neural network for segmentation of knee joint anatomy. Magn. Reson. Med..

